# Topical application of the HSP90 inhibitor 17-AAG reduces skin inflammation and partially restores microbial balance: implications for atopic dermatitis therapy

**DOI:** 10.1038/s41598-025-05307-3

**Published:** 2025-07-01

**Authors:** Krzysztof Sitko, Ewa Piotrowska, Magdalena Podlacha, Natalia Zagórska, Michał D. Starke, Magdalena Trzeciak, Stefan Tukaj

**Affiliations:** 1 Department of Molecular Biology, Faculty of Biology, University of Gdańsk, Wita Stwosza 59, 80-308 Gdańsk, Poland; 2 Department of Plant Experimental Biology and Biotechnology, Faculty of Biology, University of Gdańsk, Wita Stwosza 59, 80-308 Gdańsk, Poland; 3 Department of Dermatology, Venereology and Allergology, University Clinical Center in Gdańsk, Gdańsk, Poland; 4 Department of Dermatology, Venereology and Allergology, Faculty of Medicine, Medical University of Gdańsk, 80-308 Gdańsk, Poland

**Keywords:** Heat shock proteins, Mouse model, Microbiota, *Staphylococcus aureus*, Eosinophiles, Keratinocytes., Immunology, Adaptive immunity, Cytokines, Immunological disorders, Inflammation, Innate immunity

## Abstract

Heat shock proteins belonging to the HSP90 family promote inflammation and are potential therapeutic targets in inflammatory and autoimmune diseases. Here the effects of the HSP90 inhibitor 17-AAG applied topically were evaluated in a DNCB-induced murine model of atopic dermatitis (AD). The use of 17-AAG improved clinical disease activity without causing toxicity in the animals. Topical application of 17-AAG resulted in reduced epidermal hyperplasia, decreased expression of TSLP, IL-5, and IL-6, as well as reduced activation of NF-κB in the skin. In addition, the eosinophil proportion in the blood and eosinophil peroxidase (EPX) activity in the skin were significantly reduced in 17-AAG-treated AD mice. The inhibitory effects of 17-AAG on the production of epidermal alarmins, T-helper cell-associated cytokines, and ROS release were demonstrated in cultures of activated human keratinocytes, CD4^+^ T lymphocytes, and eosinophils, respectively. Finally, next-generation sequencing metagenomic approaches revealed that topical application of 17-AAG partially restored the normal gut microbiome in AD mice. Moreover, 17-AAG inhibited *Staphylococcus aureus* biofilm formation in vitro. The findings of this study, combined with the observed increase in HSP90 and EPX activity in the leukocytes of the analyzed cohort of AD patients, support the potential therapeutic use of HSP90 inhibitors in individuals with AD.

**Supplementary Information:**

The online version contains supplementary material available at 10.1038/s41598-025-05307-3.

## Introduction

Heat shock protein 90 (HSP90) is an evolutionarily conserved family of molecular chaperones involved in the stability and activation of over 300 client proteins under normal cellular conditions^1^. This family includes cytoplasmic isoforms of HSP90, comprising a constitutive form (HSP90AB) and an inducible form (HSP90AA), as well as the endoplasmic reticulum (Grp94/gp96) and the mitochondrial (TRAP1) isoforms. HSP90 functions as a crucial molecular chaperone that stabilizes oncogenic proteins, facilitating tumor growth and survival. Both intracellular and extracellular forms of HSP90 contribute to this stabilization, raising interest in HSP90 inhibitors as potential cancer therapies. Advanced clinical studies are currently assessing the efficacy of these inhibitors across various cancer types^2^. HSP90’s chaperone function is linked to its ability to bind and hydrolyze ATP, a process finely regulated by co-chaperones and post-translational modifications, including acetylation^3^. The N-terminal domain of HSP90 contains a nucleotide-binding pocket that serves as the primary binding site for most HSP90 inhibitors. HSP90 inhibition can disrupt pathways such as JAK-STAT and suppress the activation of NF-κB, which are implicated in various cellular processes, including proliferation, differentiation, and inflammation^4^, making this therapy particularly attractive for inflammatory and autoimmune diseases^5,6^. The effectiveness of this therapy has been thoroughly demonstrated in preclinical models of epidermolysis bullosa acquisita (EBA), an autoimmune blistering skin disorder^7–9^. We were also the first to highlight the potential role of both extracellular and intracellular HSP90 in the development of atopic dermatitis (AD)^10,11^, a common non-communicable inflammatory skin disease characterized by recurring eczematous lesions and intense itching^12^. Additionally, recent preliminary evidence from proof-of-concept studies suggests that the orally administered HSP90 inhibitor RGRN-305 leads to significant improvements in psoriasis and shows promise in randomized trials for hidradenitis suppurativa^13,14^. To date, there have been no studies on the use of HSP90 inhibitors in the treatment of patients with AD.

Our study examined the effects of the topically applied HSP90 inhibitor, 17-AAG, on clinical parameters, immune response, microbiome composition, and toxicity in a mouse model of 2,4-dinitrochlorobenzene (DNCB)-induced human-like AD which is widely used to assess novel treatment strategies and compounds^15^. Additionally, we assessed the significance of HSP90 concerning its expression and activity in tape strip skin samples and blood leukocytes obtained from AD patients, respectively. Finally, we utilized *Staphylococcus aureus* strains and human cell cultures to confirm the effects observed in mice and further elucidate the mechanisms underlying anti-HSP90 therapy. Through this research, we aimed to contribute to the development of innovative, safe, and effective therapeutic strategies for managing atopic dermatitis and related conditions.

## Results

### Topical 17-AAG treatment reduces disease activity in an experimental mouse model of 2,4-dinitrochlorobenzene (DNCB)-induced human-like atopic dermatitis

**Fig. 1:**
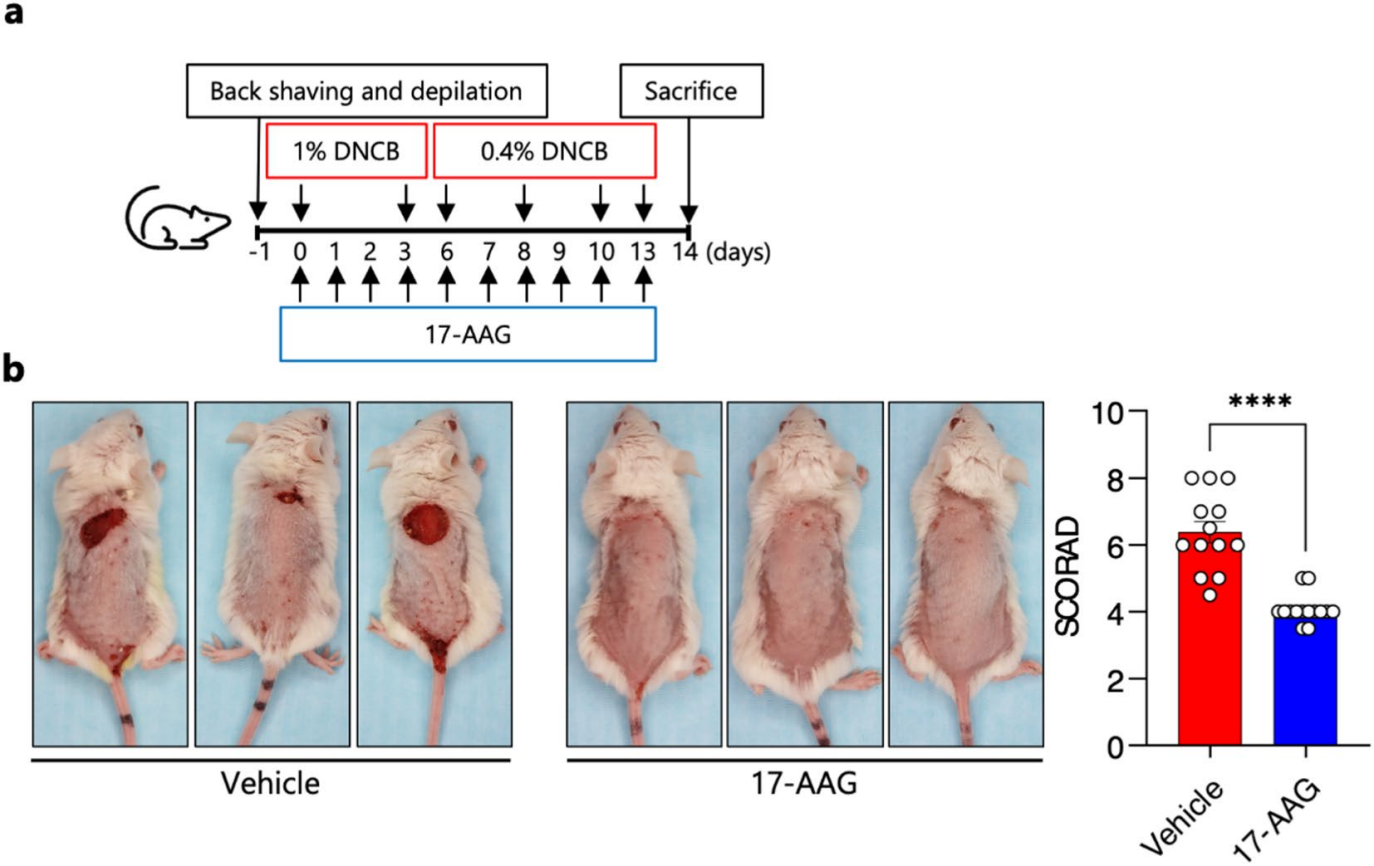
Topical 17-AAG treatment reduces disease activity in an experimental mouse model of DNCB-induced human-like AD. (**a**) Schematic representation of experimental design. AD-like skin inflammation was induced in female BALB/c mice by dinitrochlorobenzene (DNCB) application to the shaved and depilated back skin (day − 1) as follows: 1% DNCB on days 0 and 3, followed by 0.4% DNCB on days 6, 8, 10 and 13. 17-AAG (0.5 µM) or vehicle (DMSO: acetone, 1:40 vol/vol) was applied each day with a 2-h interval to DNCB. (**b**) Representative images of vehicle- and 17-AAG-treated mice at the end of the 14-day treatment period and clinical disease severity of vehicle and 17-AAG-treated mice represented as cumulative SCORAD index. Data are expressed as mean ± SEM (with individual values) of three independent experiments. **** *P* < 0.0001.

Topically applied 17-AAG (0.5 µM) (Fig. 1a) significantly reduced the development of characteristic clinical symptoms of AD (i.e., erythema, edema, excoriation, and scaling/dryness) expressed as modified SCORAD index compared with vehicle-treated animals (Fig. 1b). Clinical efficacy of 17-AAG was observed in a dose-dependent manner in an additional independent experiment (Fig. S1). Of note, therapy with topically administered 17-AAG on shaved back skin (6 cm^2^) at a dose of 0.5 µM per day for two weeks was safe, as it did not result in mortality, skin irritation or a decrease in body weight of the animals. Furthermore, the therapy did not induce systemic toxicity, as the parameters of peripheral blood morphology (Table S1) and serum biochemical markers (Table S2) were not significantly altered, except for the inhibitory effect of 17-AAG on the percentage of circulating eosinophils.

### Topical application of 17-AAG influences the type 2 immune response in mice with atopic dermatitis

**Fig. 2:**
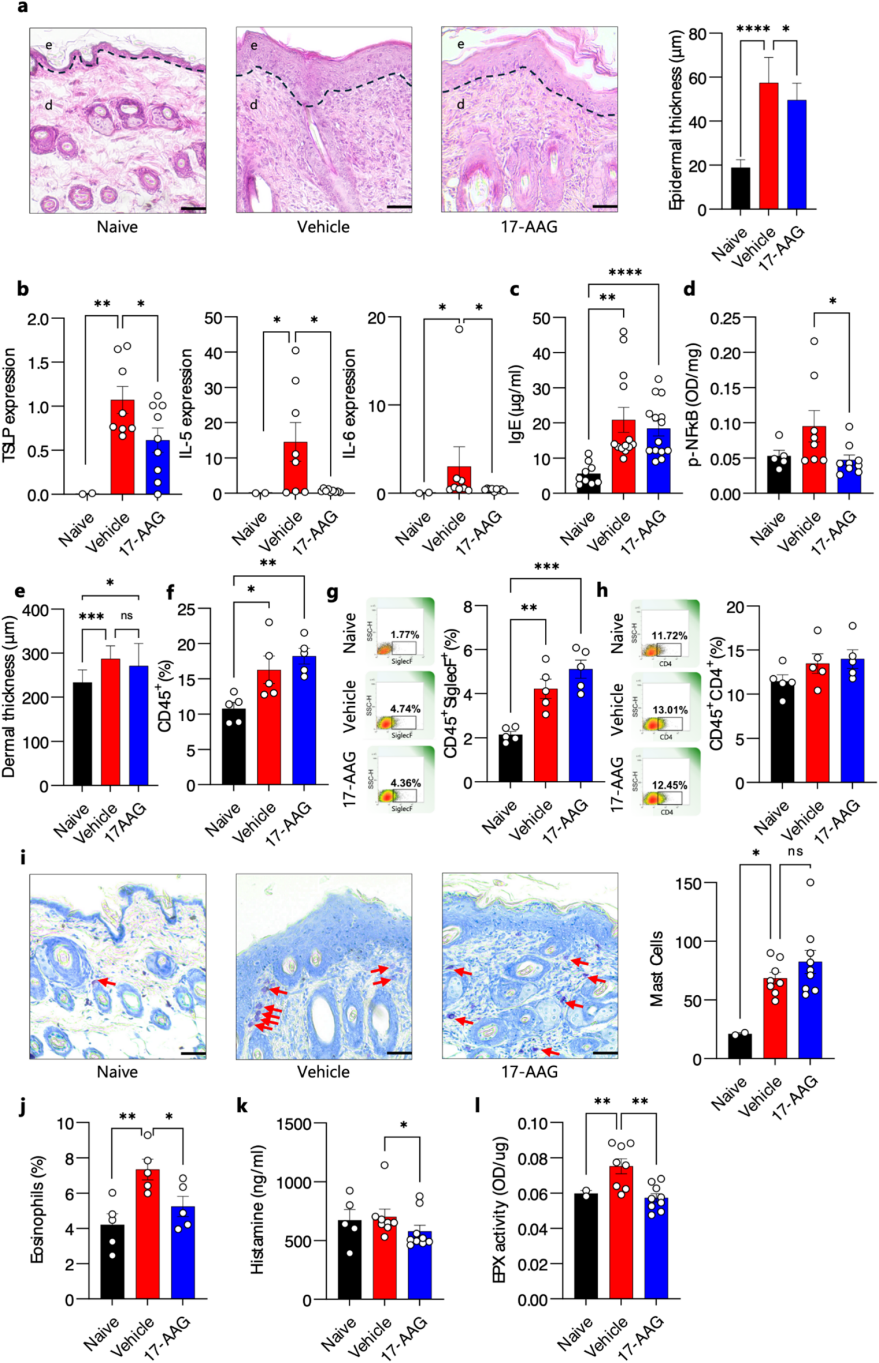
Topical Application of 17-AAG Influences the Type 2 Immune Response in Mice with AD. (**a**) H&E- stained skin sections of naïve, vehicle- and 17-AAG-treated mice. Bars = 50 μm. (**b**) Relative expression of TSLP, IL-5, and IL-6 in the biopsies of mouse back skin, by qPCR. ELISA test results of (**c**) IgE level in the serum of mice and (**d**) NF-κB phosphorylation in the biopsies of mouse back skin. (**e**) Histological skin infiltration, as well as **(f**) the number of infiltrating leukocytes (CD45^+^), including (**g**) eosinophils (CD45^+^Siglec-F^+^) and **(h**) T helper cells (CD45^+^CD4^+^), were assessed in dissociated skin biopsies using flow cytometry. (**i**) Toluidine blue- stained skin sections of naïve, vehicle- and 17-AAG-treated mice. Bars = 50 μm. (**j**) Eosinophil proportion (% of WBC) in blood via hematology analyzer. (**k**) Histamine level in mouse serum, by ELISA. (**l**) Eosinophil peroxidase (EPX) activity, as measured by OPD assay in the back skin biopsies. Data are expressed as mean ± SEM (with individual values) or mean ± SD (in the case of epidermal thickness) of three independent experiments. * *P* < 0.05, ** *P* < 0.01, *** *P* < 0.001, **** *P* < 0.0001; ns, no significance; H&E, Hematoxylin and Eosin; OPD, o-phenylenediamine dihydrochloride; OD, optical density; WBC, white blood cells; e, epidermis; d, dermis.

The topical application of 17-AAG resulted in the reduction of epidermal hyperplasia (Fig. 2a) and a decreased expression of TSLP, IL-5, and IL-6 in skin biopsies (Fig. 2b). The expression of other analyzed cytokines, including IL-4, IL-31RA, TNF-α, IL-33, IL-1β, IL-10, IL-13, and IFN-γ, was not significantly altered by the treatment; however, some inhibitory trends were observed (Fig. S2). Statistically insignificant tendency toward a decrease in circulating IgE levels was also noted (Fig. 2c). Our findings indicate that the topical application of 17-AAG effectively reduced NF-κB activation in the skin (Fig. 2d). Although the number of infiltrating leukocytes (Fig. 2e, f) including eosinophils (Fig. 2g), CD4^+^ T cells (Fig. 2h), and mast cells (Fig. 2i) in the skin was not significantly affected by the topical application of 17-AAG, circulating eosinophil percentages (Fig. 2j), serum levels of histamine (Fig. 2k), and eosinophil peroxidase (EPX) activity (Fig. 2l) in the skin were significantly reduced in 17-AAG-treated AD mice compared to vehicle-treated AD mice.

### 17-AAG inhibits epidermal IL-33 alarmin, T-helper cell-associated cytokines, and reactive oxygen species in cultures of activated human keratinocytes, CD4^+^ T lymphocytes, and eosinophils, respectively

**Fig. 3:**
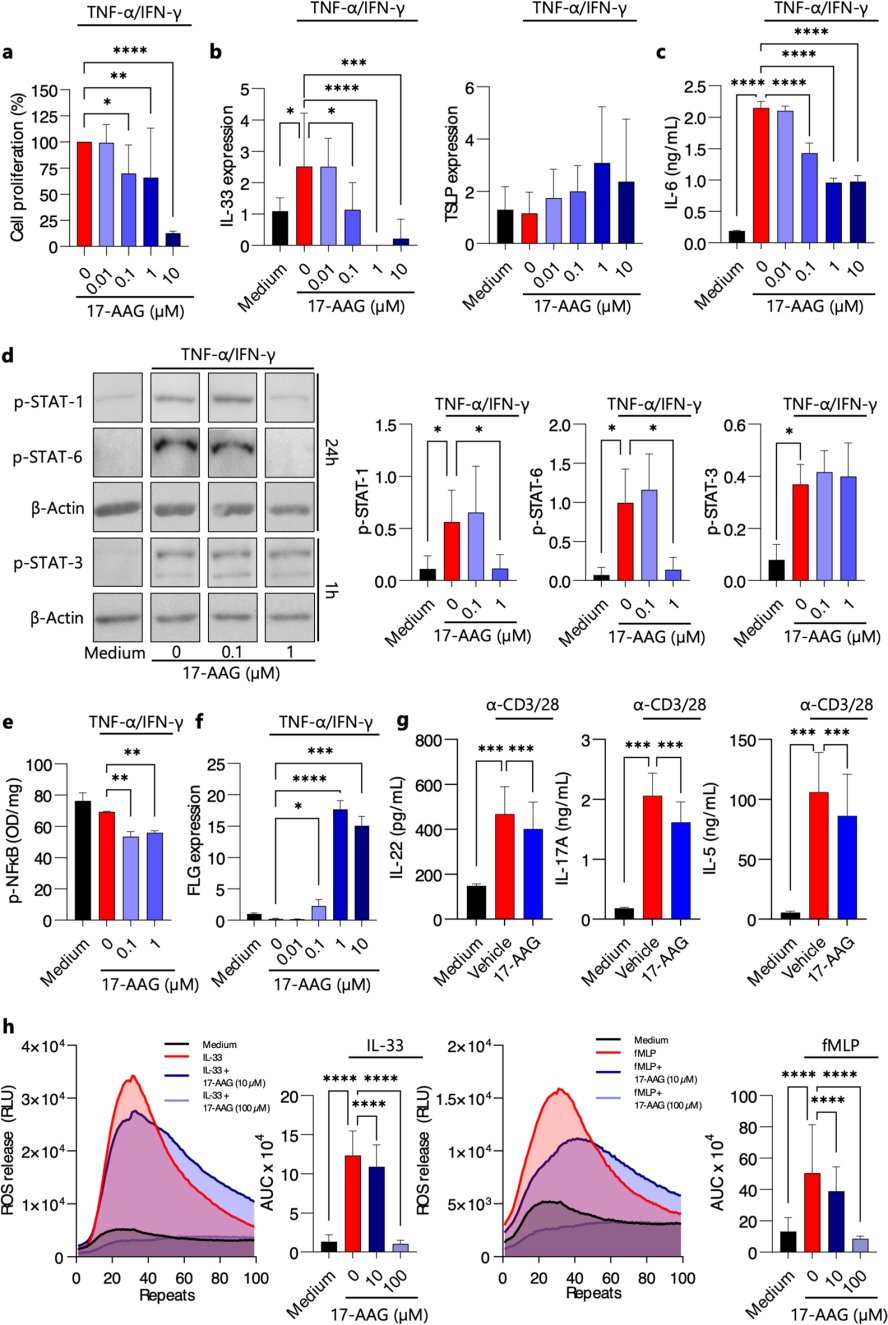
17-AAG inhibits the expression of IL-33, the secretion of IL-6, T-helper cell-associated cytokines, and reactive oxygen species in cultures of activated human keratinocytes, CD4 + T lymphocytes, and eosinophils, respectively. TNF-α/IFN-γ- stimulated human keratinocytes (HaCaT) cells were cultured in presence of DMSO 0.1% (Vehicle) or various doses of 17-AAG. (**a**) Cell proliferation ELISA results as BrdU incorporation (%) after 6 h of incubation. (**b**) Relative expression of IL-33 and TSLP, by qPCR. (**c**) IL-6 levels in culture supernatant, by ELISA. (**d**) Phosphorylated STAT-1, STAT-3, and STAT-6 protein levels, corrected for β-Actin, were analyzed in HaCaT cell lysates following 1-hour or 24-hour activation using Western blot. Representative bands are presented. (**e**) NF-κB phosphorylation measured by ELISA. (**f**) FLG relative expression analyzed by qPCR. (**g**) IL-5, IL-17 A, and IL-22 secretion in the culture supernatant of anti-CD3/CD28 antibody-stimulated (1 µg/mL) CD4^+^ T cells derived from healthy donors, assessed by ELISA. (**h**) Luminol-enhanced ROS release in eosinophils derived from healthy donors, activated with fMLP (2 µM) or IL-33 (100 ng/mL). Data are presented as mean ± SD. * *P* < 0.05, ** *P* < 0.01, *** *P* < 0.001, **** *P* < 0.0001; ns, no significance; BrdU, BromodeoxyUridine; RLU, Relative Light Unit; AUC, Area Under Curve; ROS, Reactive Oxygen Species; OD, optical density.

Using a TNF-α/IFN-γ-stimulated immortalized human keratinocyte line (HaCaT cells), we found that HSP90 inhibition with non-toxic concentrations of 17-AAG significantly reduced cell proliferation (Fig. 3a), decreased IL-33 expression (but not TSLP) (Fig. 3b), and inhibited the secretion of pro-inflammatory IL-6 (Fig. 3c). These effects were associated with inhibition of STAT-1 and STAT-6 signaling (Fig. 3d), attenuation of NF-κB activity (Fig. 3e), and upregulation of filament aggregating protein filaggrin (FLG) expression (Fig. 3f).

Additionally, the effect of 17-AAG on the cytokines produced by subpopulations of T helper cells was examined. Using anti-CD3/28 antibody-stimulated human CD4^+^ T cell cultures, we observed that 17-AAG significantly blocked the secretion of IL-5, IL-17 A, and IL-22 (Fig. 3g), cytokines characteristic of Th2, Th17, and Th22 cell populations, respectively. Finally, using IL-33- or fMLP-activated human eosinophils, we found that 17-AAG dose-dependently inhibited reactive oxygen species (ROS) production (Fig. 3h), one of the indicators of eosinophil activation status^16^.

### Topical application of 17-AAG impacts both skin and gut microbiota in a mouse model of atopic dermatitis

**Fig. 4:**
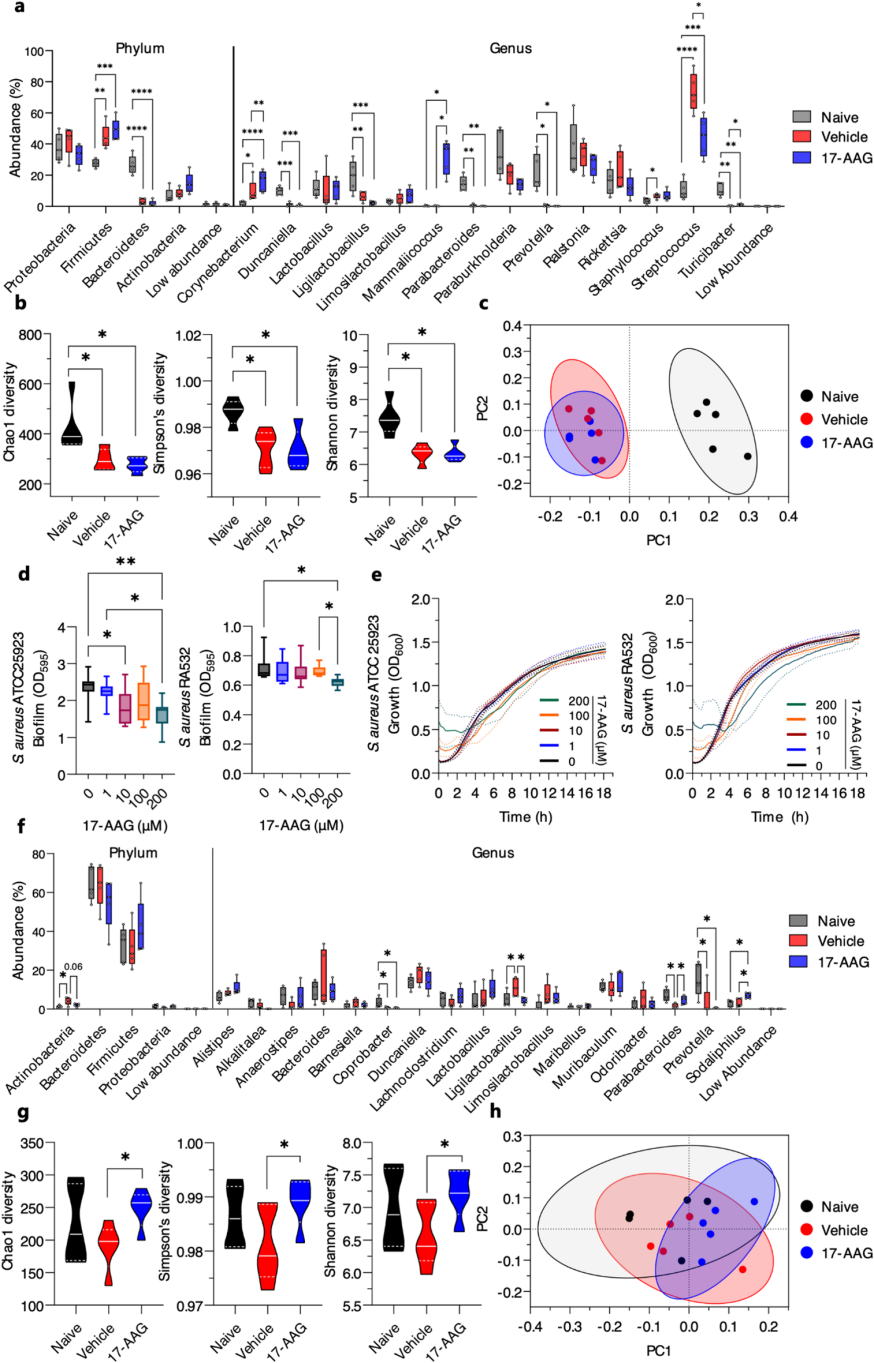
Topical Application of 17-AAG Restores Both Skin and Gut Microbiota in a Mouse Model of AD. (**a**) Relative abundances of identified phyla and genera in back skin microbiome samples from naïve, vehicle-, and 17-AAG-treated AD mice, with (**b**) alpha diversity indices (Shannon, Chao1, and Simpson’s) calculated and (**c**) weighted UniFrac PCoA analysis presented. (**d**) Effects of 17-AAG on biofilm formation and (**e**) growth rate of *S. aureus* ATCC 25,923 and RA532, expressed as min-max range and mean ± SD, respectively. (**f**) Relative abundances of identified phyla and genera in gut microbiome samples from naïve, vehicle-treated, and 17-AAG-treated AD mice, with (**g**) alpha diversity indices (Shannon, Chao1, and Simpson’s) calculated and (**h**) weighted UniFrac PCoA analysis presented. Data expressed as mean (min-max range) or mean ± SD, respectively. Taxa with mean abundances < 1% across all groups are aggregated as “Low abundance.” * *P* < 0.05, ** *P* < 0.01, *** *P* < 0.001, **** *P* < 0.0001; OD, optical density.

We profiled the microbiome of either back skin or fecal samples from co-housed littermates naïve, as well as vehicle - or 17-AAG-treated AD mice by next-generation sequencing (NGS) of the V3–V4 region of the 16 S rRNA gene. Our study revealed that in all analyzed groups, *Proteobacteria*, *Firmicutes*, *Bacteroidetes*, and *Actinobacteria* were the most abundant phyla in the skin microbiome (Fig. S3). While the relative abundances of *Proteobacteria* and *Actinobacteria* were unaffected by the topical application of DNCB or DNCB along with 17-AAG compared to naïve mice, both vehicle- and 17-AAG-treated AD mice showed significantly increased levels of *Firmicutes* and decreased levels of *Bacteroidetes* compared to naïve mice (Fig. 4a). Additionally, the microbiota diversity analysis in the samples revealed that both vehicle- and 17-AAG-treated AD mice exhibited significantly reduced alpha diversity compared to naïve mice, as indicated by the Chao1, Simpson, and Shannon indices, however no significant effect of 17-AAG therapy was observed (Fig. 4b). Similarly, DNCB sensitization significantly affected skin beta diversity, with no impact of 17-AAG treatment observed, as shown by weighted UniFrac PCoA (Fig. 4c). At the genus level, both vehicle- and 17-AAG-treated AD mice showed a significant increase in *Corynebacterium* and *Streptococcus* abundances, along with a decrease in *Duncaniella*, *Ligilactobacillus*, *Parabacteroides*, *Prevotella*, and *Turicibacter* compared to naïve mice (Fig. 4a). Additionally, 17-AAG treatment amplified *Corynebacterium* and *Mammaliicoccus* levels relative to vehicle-treated AD mice. The elevated abundance of *Streptococcus* induced by DNCB sensitization was significantly reversed in AD mice treated with 17-AAG. Likewise, the reduction in *Turicibacter* levels in AD mice was partially restored following 17-AAG treatment (Fig. 4a). We observed a significant increase in the abundance of *Staphylococcus* in AD mice. However, 17-AAG therapy showed no significant effect on *Staphylococcus* counts, including *S. aureus*. Consequently, an in vitro analysis was conducted. Our findings indicated that 17-AAG inhibits biofilm formation of two strains of *S. aureus*, i.e., methicillin-sensitive ATCC25923 and methicillin-resistant RA532, in a dose-dependent manner (Fig. 4d), without affecting their growth rate (Fig. 4e).

In the analysis of the gut microbiome, the same animals were included. Our findings revealed that *Bacteroidetes* and *Firmicutes* were the most abundant phyla across all groups (Fig. S4). We observed a notable increase in *Actinobacteria* abundance in the vehicle-treated AD mice compared to naïve mice, while 17-AAG treatment tended to restore its levels (*P* = 0.06) (Fig. 4f). Vehicle-treated AD mice had on average, lower alpha diversity than the naïve group; however, the effect did not reach significance (*P* = 0.2). Conversely, 17-AAG treatment significantly improved alpha diversity, as indicated by Chao1, Simpson, and Shannon indices (Fig. 4g). In addition, weighted UniFrac PCoA analysis showed significant differences between naïve and 17-AAG-treated AD mice with 30% of the variation explained (Fig. 4h).

At the genus level, we observed that DNCB sensitization significantly reduced the abundances of *Coprobacter*, *Parabacteroides*, and *Prevotella*, along with an increase in *Ligilactobacillus* compared to naïve mice. 17-AAG increased *Sodaliphilus* abundances compared to the vehicle-treated AD mice; however, its role in the skin disorders is currently unknown. Of note, topical application of 17-AAG significantly restored the levels of *Ligilactobacillus* and *Parabacteroides* to levels observed in the naïve group (Fig. 4f).

### Therapeutic treatment with 17-AAG reduces the severity of the experimental AD-like model

**Fig. 5:**
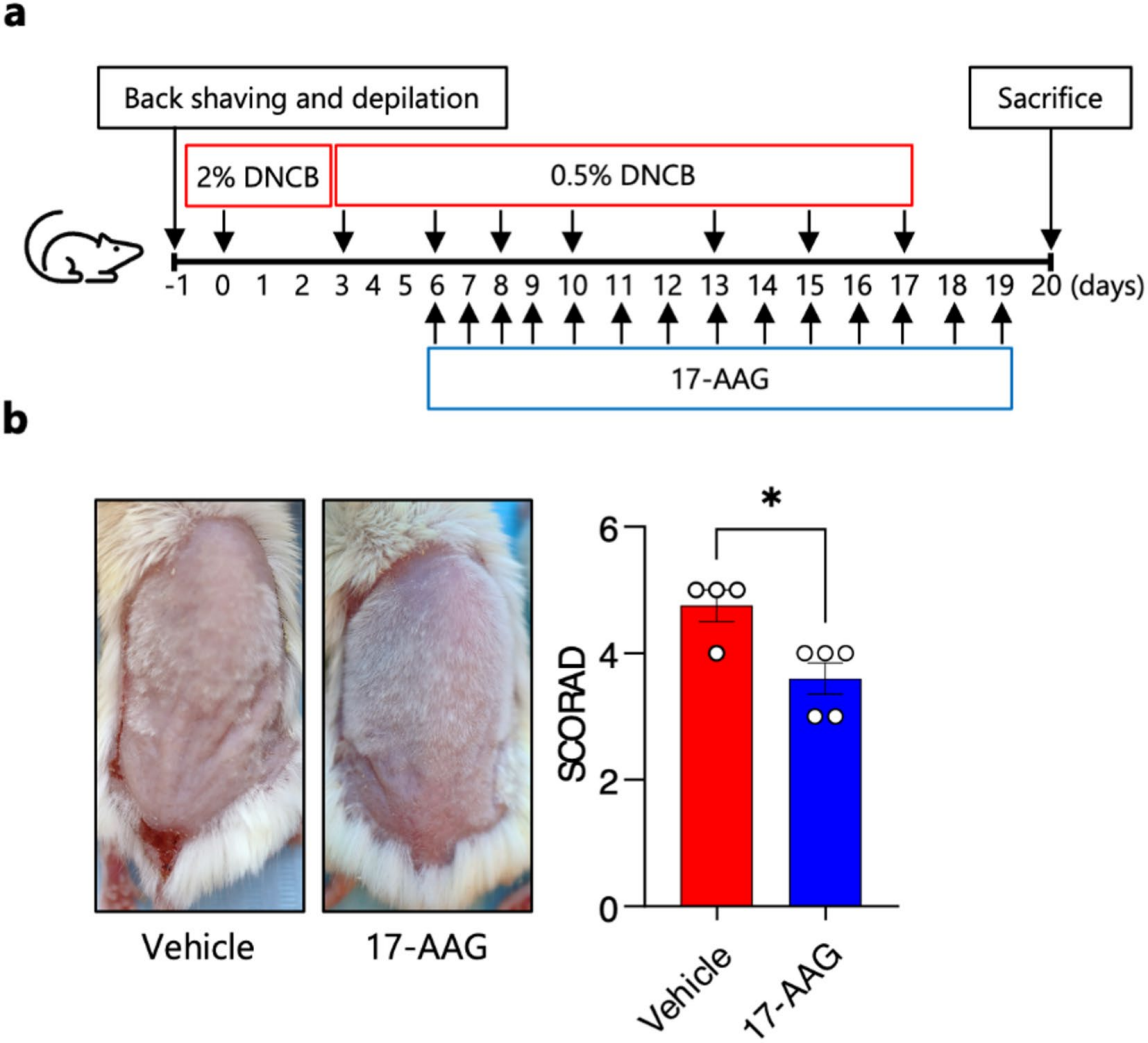
Therapeutic treatment with topically applied 17-AAG reduces disease activity in an experimental mouse model of DNCB-induced human-like AD. (**a**) Schematic representation of the experimental design. AD-like skin inflammation was induced in female BALB/c mice by applying dinitrochlorobenzene (DNCB) to the shaved and depilated back skin (day − 1) as follows: 2% DNCB on day 0, followed by 0.5% DNCB on days 3, 6, 8, 10, 13, 15 and 17. 17-AAG (0.1 µM) or vehicle (DMSO: acetone, 1:40 vol/vol) was applied daily, with a 2-hour interval following DNCB application. (**b**) Representative images of vehicle- and 17-AAG-treated mice, and clinical disease severity, represented by the cumulative SCORAD index at day 20. Data are expressed as mean ± SEM (with individual values) from one experiment. * *P* < 0.05.

We evaluated the therapeutic potential of 17-AAG in mice with already established clinical AD, induced by repetitive application of DNCB. Starting on day 6, mice were treated topically with either 17-AAG or vehicle, and their condition was monitored until day 20 (Fig. 5a). Compared to vehicle-treated controls, mice receiving 17-AAG showed a clinically significant reduction in the severity of AD-like symptoms, as indicated by the modified SCORAD index (Fig. 5b), further supporting the important role of Hsp90 in the progression of experimental AD and highlighting the therapeutic potential of topically applied 17-AAG.

### The activity of HSP90 and eosinophil peroxidase is elevated in the leukocytes of individuals with AD

**Fig. 6:**
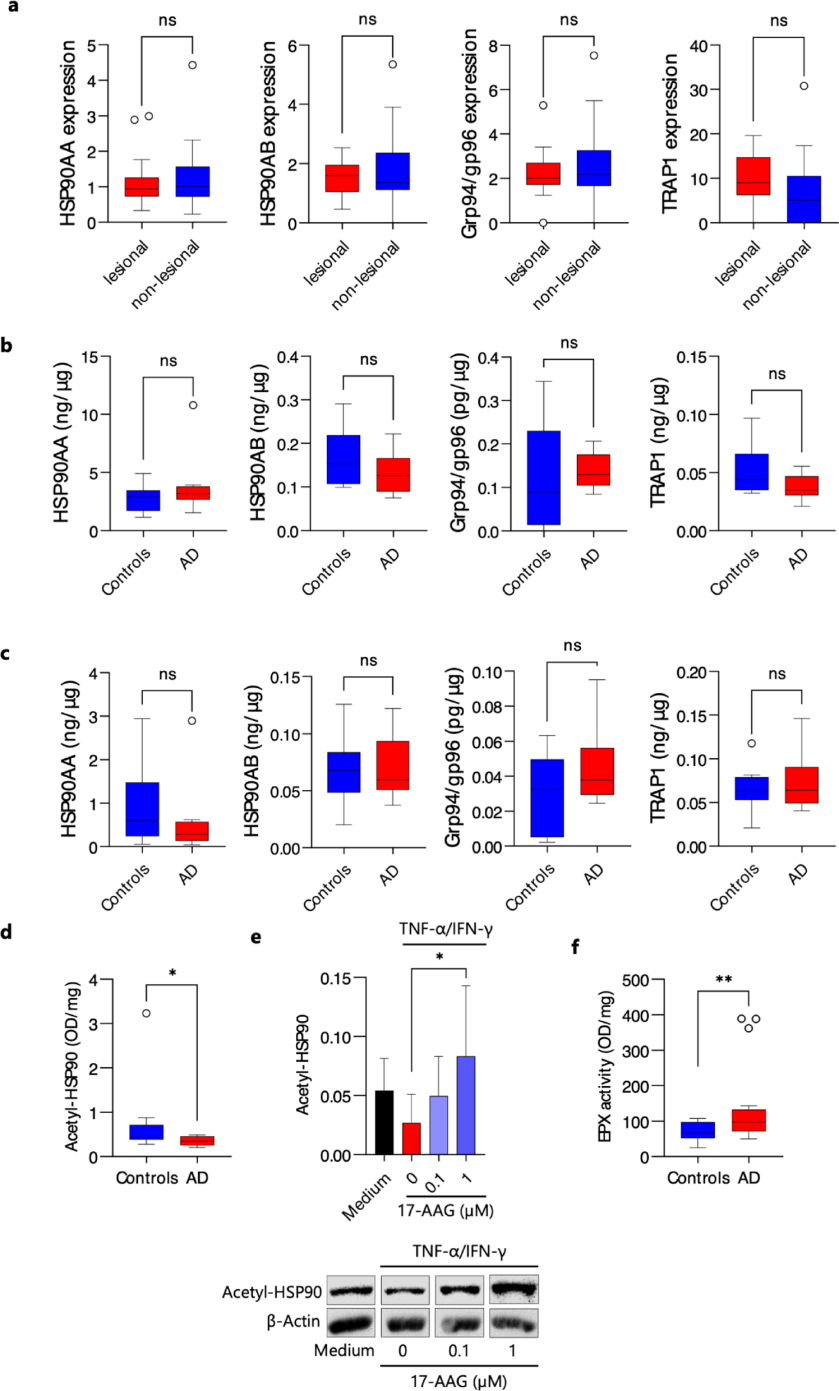
Elevated Activity of HSP90 and EPX in Leukocytes of Individuals with AD. (**a**) Relative HSP90 homolog expression measured in duplicate from 10 consecutive tape strips sampled from lesional and non-lesional skin of patients with AD. Data are expressed as mean ± SEM (with individual values). (**b**) HSP90 isoform level relative to total protein content of peripheral blood mononuclear cells. (**c**) HSP90 isoform level relative to total protein content of polymorphonuclear leukocytes. Both cell populations were derived from patients with AD and age- and gender-matched healthy individuals. Data are expressed as box-and-whisker plots (Tukey). (**d**) ELISA results showing acetyl-HSP90AA levels in PBMCs from AD patients compared to age- and gender-matched healthy controls. (**e**) β-Actin-corrected levels of acetyl-HSP90 in activated (TNF-α/IFN-γ, 10 ng/mL) human keratinocyte (HaCaT) cells treated with 0.1% DMSO or non-toxic concentrations of 17-AAG, analyzed by Western blot with representative bands presented. (**f**) Eosinophil peroxidase (EPX) activity in PMNs from AD patients and age- and gender-matched healthy controls. Data are expressed as box-and-whisker plots (Tukey) or mean ± SD. * *P* < 0.05, ** *P* < 0.01. ns, no significance.

We analyzed the gene expression of four HSP90 homologs (i.e., HSP90AA, HSP90AB, Grp94/gp96, and TRAP1) using tape-strip samples collected from 19 patients with AD, from both non-lesional and lesional skin areas. The characteristics of patients with AD are presented in the Table S3. We did not observe significant changes in the expression of the analyzed HSP90 homologs between lesional and non-lesional skin samples (Fig. 6a). Similarly, there were no significant differences in the level of HSP90 protein isoforms in polymorphonuclear leukocytes (PMNs) (Fig. 6b) and peripheral blood mononuclear cells (PBMCs) (Fig. 6c) between patients with AD (*n* = 8) and age- and gender-matched healthy individuals (*n* = 9).

Further, we determined the level of HSP90 acetylation (acetyl-HSP90) in leukocytes from patients with AD (*n* = 8) and age- and gender-matched healthy controls (*n* = 9). Notably, we observed a significantly lower level of acetyl-HSP90 in the PBMCs of individuals with AD compared to healthy controls (Fig. 6d) and that the presence of 17-AAG in cultures of human keratinocytes (HaCaT) led to increased levels of acetyl-HSP90 (Fig. 6e). Finally, we found elevated eosinophil peroxidase (EPX) activity in the PMNs of individuals with AD compared to healthy controls (Fig. 6f).

## Discussion

Our study examined the effects of the topically applied HSP90 inhibitor, 17-AAG, on clinical parameters, immune response, microbiome composition, and toxicity in a mouse model of 2,4-dinitrochlorobenzene (DNCB)-induced human-like AD which is widely used to assess novel treatment strategies and compounds^15^. Topically applied 17-AAG significantly reduced the development of characteristic clinical symptoms of AD in both prophylactic and therapeutic settings. The prophylactic treatment did not induce toxicity, as the parameters of peripheral blood morphology and serum biochemical markers were not significantly altered, except for the inhibitory effect of 17-AAG on the percentage of circulating eosinophils. The latter effect was desirable given the frequent occurrence of eosinophilia in patients with AD^17^ and the elevated eosinophil levels in mice with AD. The safety analysis of such therapy is particularly important because, although the majority of discovered HSP90 inhibitors targeting the ATP-binding pocket in the N-terminal domain of HSP90 have entered oncological clinical trials (e.g., 17-AAG) as systemic interventions, none of them was approved by the FDA due to their toxicity^18^. The results of our study suggest that the topical delivery of HSP90 inhibitors provides the advantage of reducing the risk of systemic adverse effects associated with orally or intravenously administered HSP90 inhibitors.

The pathophysiology of AD is characterized by a dysregulated immune response, predominantly skewed towards a type-2 phenotype. This response is driven by the release of epidermal alarmins, such as TSLP and IL-33, in reaction to allergens and various external stressors. The activation of T-helper 2 (Th2) cells subsequently stimulates the production of cytokines, including IL-4, IL-5, and IL-13, promoting B cell activation, specific IgE production, and the recruitment/activation of eosinophils and mast cells^12,19^. In this study, the topical application of 17-AAG resulted in the reduction of epidermal hyperplasia and a decreased expression of TSLP, IL-5, and IL-6 in skin biopsies. Previous studies have established that NF-κB mediates the induction of pro-inflammatory cytokines, such as TSLP and IL-6^20^. Given the significant role of NF-κB in the pathogenesis of AD^21^, there has been an effort to develop topical therapies targeting the NF-κB pathway^22,23^. Our findings indicate that the topical application of 17-AAG effectively reduced NF-κB activation in the skin. Furthermore, although the number of infiltrating eosinophils, CD4^+^ T cells and mast cells in the skin was not significantly affected by the topical application of 17-AAG, circulating eosinophil percentages, serum levels of histamine, and eosinophil peroxidase (EPX) activity in the skin were significantly reduced in 17-AAG-treated AD mice compared to vehicle-treated AD mice. There is an apparent discrepancy between IL-5 expression and the number of tissue-infiltrating eosinophils. Possible explanations for this include the involvement of IL-5–independent chemotactic factors, the persistence of pre-existing eosinophils in the tissue, or the functional inactivation of eosinophils despite their presence in the skin. Therefore, it seems that the reduction in cell activity through an NF-κB-dependent mechanism, rather than cell recruitment, may underlie the therapeutic effect of 17-AAG in reducing inflammatory responses in AD.

To further elucidate the potential molecular and cellular pathways related to AD that are targeted by HSP90 inhibition in vivo, we utilized activated human keratinocytes, CD4^+^ T lymphocytes, and eosinophil cell cultures. Using a TNF-α/IFN-γ-stimulated immortalized human keratinocyte line (HaCaT cells), we found that HSP90 inhibition with non-toxic concentrations of 17-AAG significantly reduced cell proliferation, decreased IL-33 expression, and inhibited the secretion of pro-inflammatory IL-6. These effects were associated with inhibition of STAT-1 and STAT-6 signaling, attenuation of NF-κB activity, and upregulation of FLG expression. The results concerning the effects of HSP90 inhibition on signaling pathways involving STAT and NF-κB are consistent with previous reports that emphasize their roles in various cellular processes, including proliferation and inflammation^4^. The positive effect of the therapy on FLG expression is also beneficial in the context of the pathophysiology of AD, as some patients with AD have an acquired defect in its expression^24^.

Because the pathogenesis of AD also involves an imbalanced adaptive immune system characterized by enhanced activation of Th2, Th17, and Th22 cell population^25^, the impact of 17-AAG on the cytokines produced by these cell subpopulations was investigated. Using anti-CD3/28 antibody-stimulated human CD4^+^ T cell cultures, we observed that 17-AAG significantly blocked the secretion of IL-5, IL-17 A, and IL-22, cytokines characteristic of Th2, Th17, and Th22 cell populations, respectively. Finally, using IL-33- or fMLP-activated human eosinophils, we found that 17-AAG dose-dependently inhibited ROS production, one of the indicators of eosinophil activation status^16^.

The microbiome plays a crucial role in the development and maintenance of immune system homeostasis. Recent research has linked changes in microbial composition (known as dysbiosis) in the skin and gut to altered immune responses and the development of AD^26^. Here, we profiled the microbiome of either back skin or fecal samples from co-housed littermates naïve, as well as vehicle - or 17-AAG-treated AD mice by NGS of the V3–V4 region of the 16 S rRNA gene. Our study revealed that in all analyzed groups, *Proteobacteria*, *Firmicutes*, *Bacteroidetes*, and *Actinobacteria* were the most abundant phyla in the skin microbiome. While the relative abundances of *Proteobacteria* and *Actinobacteria* were unaffected by the topical application of DNCB or DNCB along with 17-AAG compared to naïve mice, both vehicle- and 17-AAG-treated AD mice showed significantly increased levels of *Firmicutes* and decreased levels of *Bacteroidetes* compared to naïve mice. Additionally, the microbiota diversity analysis in the samples revealed that both vehicle- and 17-AAG-treated AD mice exhibited significantly reduced alpha diversity compared to naïve mice, as indicated by the Chao1, Simpson, and Shannon indices, however no significant effect of 17-AAG therapy was observed. Similarly, DNCB sensitization significantly affected skin beta diversity, with no impact of 17-AAG treatment observed, as shown by weighted UniFrac PCoA. At the genus level, both vehicle- and 17-AAG-treated AD mice showed a significant increase in *Corynebacterium* and *Streptococcus* abundances, along with a decrease in *Duncaniella*, *Ligilactobacillus*, *Parabacteroides*, *Prevotella*, and *Turicibacter* compared to naïve mice. Additionally, 17-AAG treatment amplified *Corynebacterium* and *Mammaliicoccus* levels relative to vehicle-treated AD mice. The elevated abundance of *Streptococcus*, a recognized risk factor for AD development^27^, induced by DNCB sensitization, was significantly reversed in AD mice treated with 17-AAG. Likewise, the reduction in *Turicibacter* levels in AD mice was partially restored following 17-AAG treatment. The increase in *Corynebacterium* abundance following topical 17-AAG treatment appears beneficial in the context of AD development and aligns with previous studies on standard AD therapies. Namely, topical applications of corticosteroids, antibiotics, and calcineurin inhibitors have been associated with enhanced microbial diversity, including colonization by *Corynebacterium*^28^. Furthermore, the expansion of *Corynebacterium* may contribute to the inhibition of *Staphylococcus aureus* (*S. aureus*) colonization on the skin^28^. We observed a significant increase in the abundance of *Staphylococcus* in AD mice. However, 17-AAG therapy showed no significant effect on *Staphylococcus* counts, including *S. aureus*, the predominant colonizer and pathogen in human AD^29^. Consequently, an in vitro analysis was conducted. Our findings indicated that 17-AAG inhibits biofilm formation of two strains of *S. aureus*, i.e., methicillin-sensitive ATCC25923 and methicillin-resistant RA532, in a dose-dependent manner, without affecting their growth rate.

In the analysis of the gut microbiome, the same animals were included. Our findings revealed that *Bacteroidetes* and *Firmicutes* were the most abundant phyla across all groups. We observed a notable increase in *Actinobacteria* abundance in the vehicle-treated AD mice compared to naïve mice, while 17-AAG treatment tended to restore its levels. Vehicle-treated AD mice had on average, lower alpha diversity than the naïve group; however, the effect did not reach significance. Conversely, 17-AAG treatment significantly improved alpha diversity, as indicated by Chao1, Simpson, and Shannon indices. In addition, weighted UniFrac PCoA analysis showed significant differences between naïve and 17-AAG-treated AD mice with 30% of the variation explained. At the genus level, we observed that DNCB sensitization significantly reduced the abundances of *Coprobacter*, *Parabacteroides*, and *Prevotella*, along with an increase in *Ligilactobacillus* compared to naïve mice. 17-AAG increased *Sodaliphilus* abundances compared to the vehicle-treated AD mice; however, its role in the skin disorders is currently unknown. Of note, topical application of 17-AAG significantly restored the levels of *Ligilactobacillus* and *Parabacteroides* to levels observed in the naïve group.

In interpreting the results of microbiome analysis in animal models, two key issues must be considered. The first concerns the natural interspecies differences that exist between the microbiota of humans and animals. The second issue is the variation in microbiome composition between different mouse models of AD and the microbiome of AD patients^30^. On the other hand, it should be emphasized that, based on our observations, the dysbiosis observed in mice treated with DNCB was partially restored by therapy with topically applied 17-AAG in the gut to levels observed in naïve mice. This comparative approach seems justified in this case. Although the exact mechanism by which topically applied 17-AAG affects the microbiome is not yet fully understood, we hypothesize that the beneficial effects of 17-AAG therapy on the microbiome may result from its direct inhibitory action on pro-inflammatory cytokine expression, *S. aureus* biofilm formation, and eosinophil count or activity. Although this is the first study on the impact of HSP90 inhibition on microbiome composition, previous research has demonstrated a link between microbiome composition and eosinophil activity^31–33^, as well as associations between *S. aureus* colonization and AD activity^29^. In addition, there are at least two non-exclusive hypotheses regarding the mechanism by which 17-AAG directly inhibits biofilm formation. First, 17-AAG inhibits the canonical function of an important molecular chaperone. Therefore, we hypothesize that targeting the bacterial HSP90 homolog, HtpG, may disrupt chaperone functions that are necessary for the production or stability of proteins involved in biofilm formation. There is evidence that 17-DMAG, another HSP90 inhibitor, disrupts *Candida albicans* biofilm formation to a degree comparable to the tetO-Hsp90/hsp90 mutant strain^34^. Additionally, blocking HSP60 with anti-HSP60 mAb effectively reduced biofilm formation in another fungus, *Histoplasma capsulatum*^35^. While evidence from bacteria is scarce, *Pseudomonas aeruginosa* DnaJ-(HSP40) deficient mutant showed significantly reduced initial adhesion and biofilm formation, likely due to the impaired pyocyanin synthesis and decreased production of extracellular DNA^36^. Moreover, HSP inhibitors (apoptozole and nonactin) in combination with chlorin-e6 associated photodynamic therapy, inhibited both mono- and multi-species biofilm formation and suppressed the vitality of Streptococci synergistically^37^. Recent studies also demonstrated the secretion of DnaK (HSP70) and GroEL (HSP60) proteins into the extracellular space during biofilm growth in *Acinetobacter baumannii*^38^. Secondly, one study showed that 17-AAG, in combination with azoles, could inhibit the formation of azole-resistant fungal biofilms 39. The same study theorised that by inhibiting ATPase activity in fungal cells, 17-AAG may also block drug efflux pumps on the fungal cell membrane^39^. Perhaps in a similar fashion, impairing energy-dependent efflux mechanisms could sensitise *S. aureus* cells and biofilms. One such target could be the NorA efflux pump, which actively expels antibiotics and exports quorum-sensing molecules, and was shown to be successfully targeted by efflux pump inhibitors, which led to biofilm inhibition^40^.

Tape strips are widely used in dermatological research as a minimally invasive method for sampling the epidermis, eliminating the need for skin biopsies^41^. In this study, we analyzed the gene expression of four HSP90 homologs using tape-strip samples collected from patients with AD, from both non-lesional and lesional skin areas. We did not observe significant changes in the expression of the analyzed HSP90 homologs between lesional and non-lesional skin samples. Similarly, there were no significant differences in the level of HSP90 protein isoforms in PMNs and PBMCs between patients with AD and age- and gender-matched healthy individuals.

The primary goal of therapies using HSP90 inhibitors, such as 17-AAG, is to inhibit the chaperone activity of HSP90, thereby disrupting the function of its client proteins^5^. In fact, the activity of HSP90 is regulated by multiple mechanisms, including post-translational modifications such as acetylation^3^. Therefore, we determined the level of HSP90 acetylation (acetyl-HSP90) in leukocytes from patients with AD and age- and gender-matched healthy controls. Notably, we observed a significantly lower level of acetyl-HSP90 in the PBMCs of individuals with AD compared to healthy controls. This reduced level of acetylation suggests a higher chaperone activity of HSP90 in AD^42^. The evidence for this relationship is the observation that the presence of 17-AAG in cultures of human keratinocytes led to increased levels of acetyl-HSP90. Finally, we found elevated eosinophil peroxidase (EPX) activity in the PMNs of individuals with AD compared to healthy controls. The latter observation underscores the role of EPX in AD^43^ and simultaneously suggests that it may be a potential therapeutic target for treating AD with 17-AAG, as demonstrated in the murine model of AD in this study.

## Conclusions

In conclusion, based on our comprehensive preclinical observations regarding the efficacy of topical HSP90 inhibitor therapy in alleviating AD symptoms, as well as its impact on the immune system and microbiome, we anticipate that clinical trials involving AD patients or related diseases will soon follow. It is important to note that therapy utilizing HSP90 inhibitors is not limited solely to diseases characterized by increased expression of HSP90 family proteins. Our research suggests that the activity of these chaperones plays a crucial role, aligning with the broader concept of anti-HSP90 therapy, which aims to reduce HSP90 activity without altering its expression levels. Our findings are highly promising for future clinical studies, as the effective dose of the 17-AAG inhibitor was proven to be completely safe. Finally, based on the results of this study, we suggest that the primary cellular target of therapy with 17-AAG are epidermal cells, as indicated by both cell culture and animal model studies. Our conclusions are supported by observations such as the reduction in epidermal thickness in response to treatment, without significant changes in skin infiltration by leukocytes, including helper T cells, mast cells, and eosinophils. However, the activity of these leukocytes was significantly inhibited, therefore it appears that topical application of 17-AAG requires further investigation to evaluate its potential as a monotherapy for managing AD.

## Limitations

Although our findings from both in vitro studies and the DNCB-induced AD model, along with the observed increase in HSP90 and EPX activity in the leukocytes of the analyzed cohort of AD patients, support the potential use of HSP90 inhibitors in treating individuals with AD, several issues need to be considered when planning future studies. Given the heterogeneous nature of AD, other mouse models (e.g., MC903-induced, house dust mite, and genetic models) that replicate the cellular and molecular aspects of the disease in different ways should be explored. The use of alternative HSP90 inhibitors and combination therapies should also be considered. Finally, based on next-generation sequencing and bioinformatic microbiome analyses, it appears that the topical application of 17-AAG partially restores the normal gut microbiome, while its effect on the skin microbiome seems limited to a few bacterial genera, rather than impact broad compositional metrics. We speculate that this intriguing result may be due to the therapy’s specific impact on the formation of *S. aureus* biofilm, which is one of the key factors contributing to the exacerbation of atopic dermatitis symptoms, or possibly due to the inhibition of peripheral inflammatory processes mediated by eosinophils, which positively influence the gut microbiome. However, further studies are needed to confirm this hypothesis.

## Materials and methods

### Patients

This study included 19 patients with active AD, consisting of 13 males and 6 females, with a mean age of 26.8 years (range: 8–54 years). Patients were recruited from the outpatient Dermatology, Venerology, and Allergology clinics at the Medical University of Gdańsk, based on the AD diagnostic criteria proposed by Hanifin and Rajka (1980). All participants were of European descent. Patients receiving systemic immunosuppressive treatment, immunotherapies, or phototherapy, as well as those with skin infections, were excluded from the study. Written informed consent was obtained from all participants or their legal guardian for underage subjects. This research was conducted in accordance with the Declaration of Helsinki and was approved by the Independent Bioethics Committee for Scientific Research at the Medical University of Gdańsk, Poland. For patient details, see **Table S3**.

### Animals

Female Balb/c mice (6–8 weeks old) were obtained from the Tri-City University Animal Facility – Research Service Center (Poland). The mice went through a handling period and were housed in a ventilated animal room with *ad libitum* access to food and tap water.

### Ethical statement for animal use

The study protocol was approved by the Animal Care and Use Committee in Bydgoszcz, Poland. All experiments were performed by qualified personnel in accordance with National Animal Care and Use Committee guidelines as specified in Polish law by The Act on the Protection of Animals (Dz.U. 1997 nr 111 poz. 724) and European law by the Act on the Protection of Animals Used for Scientific or Educational Purposes (2010/63/EU). The research facility animals were housed in complied with the requirements of the The European Act on the Protection of Animals Used for Scientific or Educational Purposes and adhered to the European Commission’s recommendations on animal welfare in scientific research.

### AD induction

The induction of AD in mice using topical application of 2,4-dinitrochlorobenzene (DNCB) followed a previously published protocol^11^. Briefly, approximately 6 cm² of back skin was shaved and depilated one day before initiating disease induction. DNCB (237329, Merck) was dissolved in an acetone: olive oil mixture (3:1 v/v), and 200 µl of a 1% DNCB solution was applied topically to the back skin on days 0 and 3. This was followed by applications of 200 µl of a 0.4% DNCB solution on days 6, 7, 10, and 13. The experiment was terminated on day 14 (Fig. 1a). Mice were euthanized according to American Veterinary Medical Association guidelines, by cervical dislocation under deep anesthesia (Ketamine-Xylazine).

### Prophylactic treatment and clinical outcome analysis

A stock solution of 17-AAG (S1141, Selleckchem) was prepared in DMSO (D2650, Sigma-Aldrich) and freshly diluted in a DMSO: acetone solvent (1:40 v/v) to a concentration of 0.5 µM. A volume of 200 µl of this prepared solution was applied to the shaved back skin of the animals (Fig. 1a). The severity of clinical symptoms was assessed on days 7 and 14 using a modified Scoring Atopic Dermatitis (SCORAD) system, which evaluates four skin parameters (i.e., erythema, edema, excoriation, and scaling/dryness), each rated on a four-point scale, yielding a maximum score of 12, as previously described^11^.

### Therapeutic treatment and clinical outcome analysis

Starting on day 6, mice were treated topically with either 17-AAG (0.1 µM) or vehicle, and their condition was monitored until day 20 (Fig. 5a). The severity of clinical symptoms was assessed on day 20 using a modified Scoring Atopic Dermatitis (SCORAD) system, as previously described^44^.

### Histopathology

Lesional skin samples were prepared for histopathological examination as previously described^9^. Briefly, biopsy specimens from the experimental animals were collected on day 14 of the experiment, fixed in 4% buffered formalin, and embedded in paraffin. Tissue Sect. (6 μm thick) were stained with hematoxylin and eosin (H&E) to evaluate general morphology, or with 0.01% toluidine blue for mast cell detection. Epidermal thickness, and mast cell count were measured using a light microscope at 100× magnification in six randomly selected areas per slide.

### Blood morphology

Blood samples were collected on day 14, prior to euthanasia of the animals. Fresh, EDTA-anticoagulated blood from naïve, vehicle- (DMSO: aceton, 1:40 v/v) or 17-AAG-treated AD mice, was analyzed using a 5160VET analyzer (URIT, China). The analysis included the number and characteristics of selected morphotic elements, as well as the percentages (%) or numbers (#) of specific cell subgroups. Table S1 provides more details.

### Biochemical safety analysis

Blood samples were collected on day 14, prior to euthanasia of the animals. Fresh, EDTA-anticoagulated blood from naïve, vehicle (DMSO: aceton, 1:40 v/v)-treated AD, and 17-AAG-treated mice, was analyzed using the Exigo C-200 analyzer (Boule Diagnostics AB, Sweden) using C200 comprehensive panel (1430002, Alpha Diagnostics). Table S2 provides more details.

### Microbiome analysis

Stool and skin swab samples were collected on day 14, prior to euthanasia, and processed by an external specialized company (Genomed S.A., Poland). Sequence analysis of the bacteria and archaea present in the samples was performed on the V3-V4 region of the 16 S rRNA gene using the 341f/785r primer pair. PCR amplification was conducted with Q5 Hot Start High-Fidelity 2X Master Mix (NEB, USA) following the manufacturer’s instructions. Sequencing was carried out on the MiSeq platform using paired-end technology (2 × 300 nt) with the v3 Illumina reagent kit (Illumina Inc., USA). Demultiplexing and FASTQ generation were performed using MiSeq Reporter (MSR v2.6, USA). Samples were analyzed using Qiime 2^45^ and Kraken 2^46^, with additional filtering to remove OTUs corresponding to mitochondria, cyanobacteria, or chloroplasts, as previously described^47^. Taxa with mean abundances < 1% across all groups were aggregated as “Low abundance”.

### Eosinophil peroxidase assay

Eosinophil peroxidase (EPX) activity was measured in mouse skin tissue extracts and human PMN extracts using a colorimetric assay on microplates, as previously described with minor modifications^48^. The assay was performed in a final volume of 125 µL per well, consisting of 75 µL of OPD substrate solution containing 50 mM Tris-HCl, pH 8, 0.1% Triton X-100, 8.8 mM H₂O₂, 6 mM KBr, and 10 mM, o-phenylenediamine dihydrochloride (P1526, Merck) combined with 50 µL of the sample diluted in PBS (14190136, Thermo Fisher Scientific). The reaction mixtures were incubated at 37 °C for 30 min, followed by the addition of 50 µL of 2 N H₂SO₄ to stop the reaction. The absorbance was measured at 490 nm using a Synergy H1 Plate Reader (BioTek, USA).

### Enzyme-linked immunosorbent and colorimetric assays

Detection of the acetylated form of HSP90 (acetyl-HSP90) was performed using an in-house ELISA. Briefly, a high-binding 96-well plate (NEST, China) was coated overnight at 4 °C with 50 µL of 2 µg/mL anti-Hsp90 antibodies (MABC1106, Merck) in 0.1 M Na-carbonate buffer (pH 9.6). All subsequent incubations were performed on an orbital shaker at 700 rpm. After blocking with Smartblock (113050, Candor) for 1 h at room temperature (RT), the plate was washed three times with PBS containing 0.05% Tween-20 (P1379, Merck). Cell extracts, diluted in blocking buffer, were then added to the plate and incubated for 2 h at RT. Following a washing step, anti-acetyl-Hsp90 antibodies (ABIN6285474, antibodies-online.com), diluted in blocking buffer, were added and incubated for 2 h at RT. After another washing step, HRP-conjugated secondary antibodies (ab205718, Abcam) were added and incubated for 1 h at RT. After washing, the substrate solution TMB (ab171522, Abcam) was added, and the reaction was stopped after 20 min with 0.5 M H₂SO₄. The absorbance was measured at 490 nm using a BioTek Synergy H1 Plate Reader (USA).

Commercially available ELISA-based kits were used following the manufacturer’s protocols. These included the Human IgE Uncoated ELISA Kit (88-50610-88, Invitrogen), Human NFkB p65 (Phospho) [pS536] InstantOne™ ELISA Kit (85-86082, Invitrogen), Cell Proliferation ELISA, BrdU (11647229001, Roche), ELISA MAX™ Deluxe Set Human IL-6 (430504, BioLegend), ELISA MAX™ Deluxe Set Human IL-17 A (433914, BioLegend), ELISA MAX™ Deluxe Set Human IL-5 (430404, BioLegend), ELISA MAX™ Deluxe Set Human IL-22 (434504, BioLegend), as well as assays for Histamine (ab285333, abcam), HSP90AA (ADI-EKS-895, Enzo Life Sciences), HSP90AB (A314298, Antibodies.com), Grp94/gp96 (A74824, Antibodies.com), TRAP1 (A313169, Antibodies.com) and Pierce BCA Protein Assay Kit (23225, Thermo Fisher Scientific).

### Tape strip analysis

Ten consecutive tape strips were collected from the same anatomical region of both lesional and non-lesional skin of AD patients and stored at − 80 °C, as previously described^49^.

### Real-time qPCR analysis

The relative expression of selected genes in tape strips from AD patients and mouse skin biopsies was analyzed as described previously, with minor modifications^11^. Total RNA was isolated from the samples using the RNeasy Mini Kit (74104, QIAGEN) and reverse-transcribed with the QuantiTect Reverse Transcription Kit (205311, QIAGEN). The resulting products were analyzed using the QuantiNova SYBR Green PCR Kit (208054, QIAGEN) on a LightCycler^®^ 480 Instrument II (Roche, Switzerland). The relative expression of selected genes was analyzed as previously described^11^. For mouse skin biopsy and tape strip analyses, three reference genes (*Gapdh*, *Actb*, and *Rpl13a*) and two reference genes (*Gapdh* and *Actb*) were chosen, respectively, using BestKeeper© software version 1.0^50^. The cycling conditions were as follows: an initial denaturation step at 95 °C for 2 min, followed by 50 cycles of denaturation at 95 °C for 5 s, annealing at 60 °C for 10 s, and a subsequent melting analysis. Each reaction was performed in duplicate, and the relative gene expression levels were analyzed using the 2 − ΔΔCq method. The primer sequences used for real-time qPCR are listed in Table S4.

### Lymphocyte isolation

Peripheral blood mononuclear cells (PBMCs) and polymorphonuclear cells (PMNs) were isolated from the venous blood of AD patients (*n* = 8) and healthy volunteers (*n* = 9) using Histopaque-1077 (H8889, Sigma-Aldrich) and Histopaque-1119 (11191, Sigma-Aldrich) gradient centrifugation, respectively, according to the manufacturer’s instructions. Human eosinophils and CD4^+^ T cells were isolated using the Eosinophil Isolation Kit (130-092-010, Miltenyi Biotec) and CD4^+^ T Cell Isolation Kit (130-096-533, Miltenyi Biotec), respectively, following the manufacturers’ protocols. The purity of the isolated eosinophils and CD4^+^ T cells was assessed by staining with anti-CD16-FITC (302006, Biolegend) and anti-CD4-FITC (300538, Biolegend) antibodies, respectively, followed by flow cytometry analysis (CyFlow Cube 6 flow cytometer, Sysmex, Germany). The purity of the isolates was estimated to be approximately 95%.

### Immune cell infiltrate analysis

Skin biopsies for flow cytometry analysis were performed as previously described with minor modifications^51^. Mice were euthanized on day 14, and lesional skin samples were collected and flash frozen in liquid nitrogen before storing in -80 °C. The frozen skin was cut into small pieces, resuspended in 2.5 mL of RPMI 1640 without phenol red (11835030, Thermo Fisher Scientific) containing 2 mg/mL type IV collagenase (C4-28-100MG, Merck) and 1 mg/mL DNase I (101041590001, Roche), and digested for 1 h at 37 °C on a magnetic stirrer. After digestion, the cell suspension was filtered through a 70 μm strainer, diluted in FACS buffer: 0.5% BSA (05482, Sigma-Aldrich), 2 mM EDTA (115932805, Chempur), and centrifuged at 2000 rpm for 10 min at 4 °C. The skin cells were then resuspended in 1 mL of FACS buffer and stained with anti-CD45 clone I3/2.3 (147712, BioLegend), anti-CD4 clone GK1.5 (100406, BioLegend), and anti-Siglec-F clone S17007L (155504, BioLegend) antibodies for 30 min at room temperature. Viable single cells were analyzed based on forward and side light scatter properties using a CyFlow Cube 6 flow cytometer (Sysmex, Germany).

### Reactive oxygen species release assay

ROS release assay was performed as described previously with minor modifications^52^. Purified eosinophils were resuspended in RPMI 1640 without phenol red (11835-030, Thermo Fisher Scientific), supplemented with 10% FBS (A3160801, Thermo Fisher Scientific), and plated onto a 96-well plate (Greiner Bio-One, Germany). The cells were pre-incubated for 1 h, without of with varying concentrations of 17-AAG. Following pre-incubation, the cells were stimulated with either 2 µM fMLP (F3506, Merck) or 100 ng/mL IL-33 (581802, Biolegend). Reactive oxygen species (ROS) release was immediately measured using luminol-enhanced chemiluminescence (123072, Merck) on an EnSpire Multimode Plate Reader (PerkinElmer, USA).

### Cell cultures

Immortalized keratinocytes (HaCaT) were cultured in high-glucose DMEM (41966-029, ThermoFisher Scientific), supplemented with 10% heat-inactivated fetal bovine serum (A3160801, ThermoFisher Scientific) and 1% penicillin-streptomycin solution (15140122, ThermoFisher Scientific) in 75 cm² cell flasks or 96-well plates. The cultures were maintained in a humidified atmosphere containing 5% CO₂ at 37 °C. HaCaT cells were stimulated with 10 µg/mL of IFN-γ and 10 µg/mL of TNF-α once they reached 70–80% confluency, and subsequently cultured in the presence or absence of 17-AAG for either 1–24 h. Purified CD4^+^ T cells were resuspended at 1 × 10⁶ cells per ml in RPMI 1640 (11835030, ThermoFisher Scientific) supplemented with 10% fetal bovine serum (A3160801, ThermoFisher Scientific) and 1% penicillin/streptomycin solution (15140122, ThermoFisher Scientific). The cells were stimulated with 1 µg/mL immobilized anti-CD3ε mAb (300414, BioLegend) and 1 µg/mL soluble anti-CD28 mAb (302914, BioLegend), and cultured in the presence or absence of 17-AAG in 24-well culture plates at 5% CO₂ and 37 °C for 72 h.

### Biofilm formation and growth of *Staphylococcus aureus*

Biofilm formation of two *Staphylococcus aureus* strains, methicillin-resistant RA532 and methicillin-sensitive ATCC25923, was assessed following a previously established protocol with minor modifications^53^. Growth curve analysis was performed as described previously with minor modifications^54^. To investigate whether 17-AAG affects biofilm formation, two strains of *S. aureus* were grown overnight at 37 °C with rotation, then diluted 1:100 in the media that in preliminary testing provided best biofilm formation for each strain i.e., LB (L3022, Merck) supplemented with 2 mg/mL glucose (G7021, Merck) and TSB (P-0120, BTL) with 10 mg/mL glucose (G7021, Merck), for ATCC25923 and RA532, respectively. Bacterial suspensions were added to a 96-well plate (701001, NEST), followed by the addition of 17-AAG. The plates were briefly mixed at 800 rpm and incubated for 72 h under stationary conditions at 25 °C. After incubation, the wells were washed with distilled water to remove any remaining planktonic cells or drug, then 0.1% crystal violet aqueous solution was added, and the plates were incubated at room temperature for 10 min, followed by another wash. Once the wells were completely dried, 33% acetic acid was added to each well, and the plate was shaken at 800 rpm for 10 min. Absorbance was measured at 595 nm using a Synergy H1 Plate Reader (BioTek, USA).

To determine whether 17-AAG affects bacterial growth, growth curve analysis was performed. Briefly, bacteria were grown overnight at 37 °C with rotation, then diluted 1:100 in LB media. Bacterial suspensions were added to a 96-well plate, and 17-AAG was introduced. The plates were placed in a Synergy H1 Plate Reader (BioTek, USA) for automated OD_600_ measurements, taken every 0.5 h over a 9-hour period. During incubation, the temperature was maintained at 37 °C, with intermittent shaking between measurements.

### Immunoblotting

The phosphorylation status of signaling proteins, i.e., STAT-1, STAT-3 and STAT-6, as well as HSP90 acetylation (acLys284/292), was analyzed in activated HaCaT cells cultured in the presence or absence of 17-AAG by immunoblotting. Equal amounts of cell lysates were separated on a polyacrylamide gel under denaturing conditions (SDS-PAGE) and transferred onto a nitrocellulose membrane (Bio-Rad). The membrane was incubated in blocking buffer (TBS) containing 3% non-fat milk, followed by incubation with primary antibodies targeting acetyl-HSP90 acLys284/292 (ABIN6285474, antibodies-online.com), p-STAT-1 (686401, BioLegend), p-STAT-3 (651001, BioLegend), p-STAT-6 (690101, BioLegend), and β-Actin (4970, Cell Signaling Technology) at a 1:1000 dilution. HRP-conjugated secondary antibodies, goat anti-mouse (405306, BioLegend) or anti-rabbit (A0545, Merck), were applied at a 1:2000 dilution. The Westar Supernova ECL substrate (XLS3,0100, Cyanagen) was used to visualize the protein bands. Protein levels, normalized to β-Actin, were quantified by densitometry using Image Lab 6.1.0 software (Bio-Rad, USA). Full-size scans of the exposure images corresponding to the blotting experiments presented in this study can be found in the supplementary materials (**Fig. S5**).

### Statistical analysis

Microbiome data processing and statistical analysis were performed using RStudio (PBC, Boston, MA) and GraphPad Prism software (GraphPad, San Diego, CA, USA). Data with non-normal distribution were analyzed using the Mann-Whitney U test, while normally distributed data were analyzed using Student’s t-test. For comparisons involving three or more groups, either the Kruskal-Wallis test with Dunn’s multiple comparisons or one-way ANOVA with Tukey’s multiple comparisons test was applied. P-values less than 0.05 were considered statistically significant. Animal research data in the study was reported in accordance with ARRIVE 2.0 guidelines.

## Electronic supplementary material

Below is the link to the electronic supplementary material.

Supplementary Material 1

Supplementary Material 2

## Data Availability

The datasets generated and analyzed during the current study are available in the Zenodo repository, https://doi.org/10.5281/zenodo.14524757.
