# Author Correction: Topical application of the HSP90 inhibitor 17-AAG reduces skin inflammation and partially restores microbial balance: implications for atopic dermatitis therapy

**DOI:** 10.1038/s41598-026-43906-w

**Published:** 2026-04-02

**Authors:** Krzysztof Sitko, Ewa Piotrowska, Magdalena Podlacha, Natalia Zagórska, Michał D. Starke, Magdalena Trzeciak, Stefan Tukaj

**Affiliations:** 1 Department of Molecular Biology, Faculty of Biology, University of Gdańsk, Wita Stwosza 59, 80-308 Gdańsk, Poland; 2 Department of Plant Experimental Biology and Biotechnology, Faculty of Biology, University of Gdańsk, Wita Stwosza 59, 80-308 Gdańsk, Poland; 3 Department of Dermatology, Venereology and Allergology, University Clinical Center in Gdańsk, Gdańsk, Poland; 4 Department of Dermatology, Venereology and Allergology, Faculty of Medicine, Medical University of Gdańsk, 80-308 Gdańsk, Poland

Correction to: *Scientific Reports* 10.1038/s41598-025-05307-3, published online 01 July 2025

The original version of the Article contained an error in Figure 3. In Figure 3D, the blot for p-STAT-1 for the 0 μM 17-AAG condition was a duplication of the blot for p-STAT-1 for the 0.1 μM 17-AAG condition.

The original Figure 3 and accompanying legend appear below.

The original Article has been corrected.

**Fig. 3:**
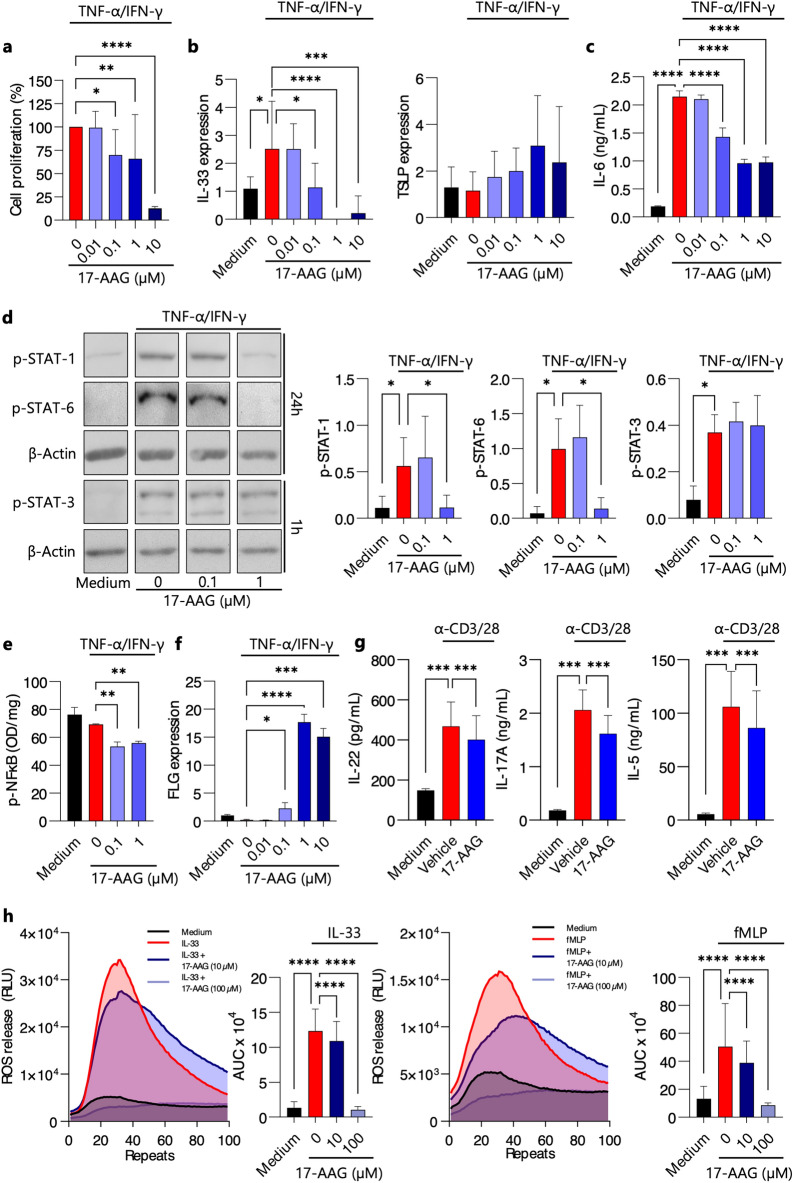
17-AAG inhibits the expression of IL-33, the secretion of IL-6, T-helper cell-associated cytokines, and reactive oxygen species in cultures of activated human keratinocytes, CD4 + T lymphocytes, and eosinophils, respectively. TNF-α/IFN-γ- stimulated human keratinocytes (HaCaT) cells were cultured in presence of DMSO 0.1% (Vehicle) or various doses of 17-AAG. (**a**) Cell proliferation ELISA results as BrdU incorporation (%) after 6 h of incubation. (**b**) Relative expression of IL-33 and TSLP, by qPCR. (**c**) IL-6 levels in culture supernatant, by ELISA. (**d**) Phosphorylated STAT-1, STAT-3, and STAT-6 protein levels, corrected for β-Actin, were analyzed in HaCaT cell lysates following 1-hour or 24-hour activation using Western blot. Representative bands are presented. (**e**) NF-κB phosphorylation measured by ELISA. (f) FLG relative expression analyzed by qPCR. (**g**) IL-5, IL-17 A, and IL-22 secretion in the culture supernatant of anti-CD3/CD28 antibody-stimulated (1 µg/mL) CD4^+^ T cells derived from healthy donors, assessed by ELISA. (**h**) Luminol-enhanced ROS release in eosinophils derived from healthy donors, activated with fMLP (2 µM) or IL-33 (100 ng/mL). Data are presented as mean ± SD. * *P* < 0.05, ** *P* < 0.01, *** *P* < 0.001, **** *P* < 0.0001; ns, no significance; BrdU, BromodeoxyUridine; RLU, Relative Light Unit; AUC, Area Under Curve; ROS, Reactive Oxygen Species; OD, optical density.

