# Supplementary material for: Topical application of the HSP90 inhibitor 17-AAG reduces skin inflammation and partially restores microbial balance: implications for atopic dermatitis therapy
Source: Sci Rep. 2025 Jul 1;15:21245. doi: 10.1038/s41598-025-05307-3 (PMC12216750; doi:10.1038/s41598-025-05307-3)
Supplement: Supplementary file 1 — Supplementary Material 1 [file 41598_2025_5307_MOESM1_ESM.docx]

**Table S1.**

| **Analyte** | **Naïve** N = 5^1^ | **Vehicle** N = 5^1^ | | **0.5 µM 17-AAG** N = 5^1^ |
| --- | --- | --- | --- | --- |
| WBC | 4.34 (4.02, 4.45) | 3.82 (3.45, 4.60) | | 3.89 (3.45, 4.38) |
| LYM% | 73 (69, 73) | ⸸52 (46, 53) | | ⸸55 (54, 55) |
| MON% | 6.08 (5.81, 6.78) | 7.99 (7.16, 8.71) | | 8.00 (6.76, 9.18) |
| NEU% | 17 (17, 18) | ⸸33 (33, 35) | | ⸸30 (30, 32) |
| EOS% | 4.48 (3.25, 4.81) | ⸸7.14 (6.43, 7.85) | | *4.87 (4.22, 6.35) |
| BASO% | 0.29 (0.21, 0.43) | 0.39 (0.35, 0.39) | | 0.50 (0.48, 0.51) |
| LYM# | 2.72 (2.59, 2.83) | ⸸1.55 (1.43, 1.67) | | ⸸1.92 (1.54, 2.02) |
| MON# | 0.23 (0.20, 0.24) | 0.26 (0.25, 0.30) | | 0.25 (0.22, 0.34) |
| NEU# | 0.71 (0.62, 0.79) | ⸸1.10 (1.03, 1.48) | | 1.06 (0.99, 1.18) |
| EOS# | 0.17 (0.10, 0.18) | 0.24 (0.20, 0.27) | | 0.17 (0.11, 0.24) |
| BASO# | 0.011 (0.009, 0.013) | 0.012 (0.010, 0.012) | | 0.018 (0.011, 0.022) |
| RBC | 7.38 (7.26, 7.55) | ⸸7.09 (6.85, 7.15) | | ⸸6.81 (6.52, 7.08) |
| HGB | 14.15 (14.10, 14.25) | ⸸13.05 (13.00, 13.20) | | ⸸13.35 (12.65, 13.45) |
| HCT | 29.55 (28.80, 30.05) | ⸸27.85 (26.85, 28.00) | | ⸸26.70 (25.30, 27.50) |
| MCV | 40.10 (39.90, 40.20) | ⸸39.25 (38.80, 39.30) | | ⸸38.95 (38.95, 39.30) |
| MCH | 19.00 (18.95, 19.40) | 18.40 (18.40, 18.90) | | 19.35 (19.15, 19.35) |
| MCHC | 47.35 (47.05, 49.00) | 47.10 (46.85, 48.95) | | 49.45 (49.00, 50.00) |
| RDW_CV | 14.30 (13.85, 14.45) | 15.05 (14.85, 15.30) | | 15.00 (14.50, 15.00) |
| PLT | 951 (884, 985) | ⸸1,144 (1,134, 1,321) | | ⸸1,295 (1,189, 1,296) |
| MPV | 5 (4.9, 5.05) | 4.9 (4.75,4.9) | | 4.95 (4.8, 4.95) |
| NRBC% | 1.56 (1.38, 1.62) | 1.83 (1.71, 2.15) | | 2.48 (2.32, 2.73) |
| NRBC# | 0.05 (0.05, 0.05) | 0.08 (0.05, 0.09) | | 0.07 (0.06, 0.10) |
| ALY% | 1.00 (0.90, 1.28) | ⸸0.59 (0.48, 0.65) | | ⸸0.53 (0.43, 0.72) |
| ALY# | 0.035 (0.030, 0.046) | ⸸0.020 (0.019, 0.023) | | ⸸0.018 (0.014, 0.024) |
| LIC% | 0.00 (0.00, 0.00) | ⸸0.33 (0.26, 0.37) | | ⸸0.37 (0.16, 0.44) |
| LIC# | 0.000 (0.000, 0.000) | ⸸0.009 (0.008, 0.016) | | ⸸0.009 (0.006, 0.016) |
| ^1^ Median (Q1, Q3); P < 0.05 vs Naive, * P < 0.05 vs Vehicle (ANOVA) | | |  |  |

White Blood Cells (WBC), Lymphocytes (LYM%/#), Monocytes (MON%/#), Neutrophils (NEU%/#), Eosinophils (EOS%/#), Basophils (BASO%/#), Red Blood Cells (RBC), Hemoglobin (HGB), Hematocrit (HCT), Mean Corpuscular Volume (MCV), Mean Corpuscular Hemoglobin (MCH), Mean Corpuscular Hemoglobin Concentration (MCHC), Red Cell Distribution Width (RDW_CV), Platelets (PLT), Mean Platelet Volume (MPV), Nucleated Red Blood Cells (NRBC%/#), Large Immature Cells (LIC%/#), and Atypical Lymphocytes (ALY). Data represents one experiment, n = 5 per group. Values are expressed as the median, with the first and third quartiles (Q1, Q3) shown in brackets. Ordinary one-way ANOVA results with p-value > 0.05 are indicated where applicable: ⸸, vs Naïve; *, vs Vehicle.

**Table S2.**

| **Analyte** | **Naïve** N = 5^1^ | **Vehicle** N = 5^1^ | **0.5 µM 17-AAG** N = 5^1^ |
| --- | --- | --- | --- |
| ALB | 28.20 (28.20, 28.70) | ⸸24.50 (23.90, 25.10) | ⸸23.80 (22.70, 24.50) |
| TP | 50.40 (50.40, 50.70) | 50.00 (49.90, 50.20) | 47.90 (47.80, 50.90) |
| GLOB | 22.30 (22.20, 23.40) | ⸸25.40 (25.00, 26.40) | ⸸25.20 (24.00, 26.40) |
| A/G | 1.27 (1.23, 1.27) | 0.97 (0.96, 0.97) | 0.94 (0.93, 0.99) |
| TB | 1.6 (1.1, 3.3) | ⸸0.1 (0.1, 0.4) | ⸸0.1 (0.1, 0.1) |
| GGT | 2 | 2 | 2 |
| AST | 85 (84, 94) | 87 (80, 107) | 84 (81, 85) |
| ALT | 23 (20, 26) | 36 (23, 40) | 27 (25, 35) |
| ALP | 5 | 5 | 5 |
| AMY | 571 (525, 576) | ⸸474 (448, 476) | *549 (513, 558) |
| CREA | 18.1 (12.0, 21.4) | 11.3 (10.1, 14.7) | 10.2 (10.0, 10.8) |
| UA | 10 (10, 10) | 15 (10, 34) | 32 (19, 39) |
| UREA | 8.37 (8.26, 8.80) | 9.85 (9.55, 10.29) | 7.95 (7.91, 9.53) |
| U/C | 439 (375, 619) | ⸸790 (675, 911) | ⸸731 (575, 1,059) |
| GLU | 10.82 (10.22, 14.16) | 8.83 (7.68, 8.88) | 7.87 (6.81, 11.03) |
| TC | 2.47 (2.34, 2.48) | 2.13 (2.09, 2.36) | 2.35 (2.15, 2.40) |
| TG | 0.88 (0.38, 1.10) | ⸸1.58 (1.48, 1.96) | 1.42 (1.08, 1.74) |
| ^1^ Median (Q1, Q3); P < 0.05 vs Naive, * P < 0.05 vs Vehicle (ANOVA) | | | |

Albumin (ALB), Alkaline Phosphatase (ALP), Alanine Aminotransferase (ALT), Aspartate Aminotransferase (AST), Amylase (AMY), Creatinine (CREA), Glucose (GLU), Gamma-Glutamyl Transferase (GGT), Total Bilirubin (TB), Cholesterol (TC), Triglycerides (TG), Total Protein (TP), Uric Acid (UA), Urea (UREA), Globulin (GLOB), Albumin/Globulin Ratio (A/G), and Urea/Creatinine Ratio (U/C). Data represents one experiment, n = 5 per group. Values are expressed as the median, with the first and third quartiles (Q1, Q3) shown in brackets. Ordinary one-way ANOVA results with p-value > 0.05 are indicated where applicable: ⸸, vs Naïve; *, vs Vehicle.

**Table S3**.

| **Patient** | **Age** | **Sex** | **Onset** | **SCORAD** | **EASI** | **Eosino-philia** | | **Comor-bidities** | **VAS** | **Sleep Score** | **Analyte** |
| --- | --- | --- | --- | --- | --- | --- | --- | --- | --- | --- | --- |
| 1 | 14 | M | 1 yo | Severe | Severe | No | | Ast. | 6 | 6 | TS, PMNs, PBMCs |
| 2 | 34 | M | Early childhood | Moderate | Moderate | No | | AR., Ast. | 7 | 7 | TS, PMNs, PBMCs |
| 3 | 11 | F | 2 yo | Severe | Severe | Yes | | AR., Ast. | 5 | 10 | TS, PMNs, PBMCs |
| 4 | 15 | F | Early childhood | Severe | Severe | No | | AR., Ast., AC. | 10 | 7 | TS, PMNs, PBMCs |
| 5 | 16 | F | Early childhood | Moderate | Moderate | Yes | | Ast. | 6 | 4 | TS |
| 6 | 13 | F | 1 yo | Severe | Severe | Yes | | AR. | 5 | 0 | TS, PMNs, PBMCs |
| 7 | 54 | M | Early childhood | Severe | Very severe | No | |  | 7 | 6 | TS, PMNs, PBMCs |
| 8 | 34 | M | 1 yo | Moderate | Moderate | Yes | | AR. | 3 | 3 | TS, PMNs, PBMCs |
| 9 | 35 | M | Early childhood | NA | Mild | Yes | |  | 3 | 3 | TS |
| 10 | 34 | M | 1 yo | Severe | Severe | NA |  | | 8 | 8 | TS, PMNs, PBMCs |
| 11 | 54 | F | 6 mo | Severe | Severe | No | | Ast. | 7 | 7 | TS |
| 12 | 8 | F | Early childhood | Severe | Severe | Yes | | AR., AC.,  CA., OAS. | 7 | 3 | TS |
| 13 | 32 | M | Early childhood | Mild | Moderate | Yes | | Ast. | 2 | 1 | TS |
| 14 | 48 | M | Early childhood | Severe | Severe | Yes | | AR. | 4 | 1 | TS |
| 15 | 14 | M | 3 mo | Severe | Severe | No | | AR., Ast. | 6 | 1 | TS |
| 16 | 34 | M | > 1 yo | Severe | Severe | No | | AR., Ast. | 8 | 8 | TS |
| 17 | 21 | M | > 1 yo | Severe | Severe | No | | AR. | 7 | 4 | TS |
| 18 | 22 | M | 7 mo | Severe | Very severe | Yes | | Ast. | 7 | 7 | TS |
| 19 | 16 | M | > 1 yo | Severe | Severe | Yes | | AR., Ast. | 8 | 8 | TS |

SCORAD cutoff values: Mild (< 25); Moderate (25–50); Severe (> 50), EASI cutoff values: Clear (0); Almost clear (0.1–1.0); Mild (1.1–7.0); Moderate (7.1–21.0); Severe (21.1–50.0); Very severe (50.1–72.0), Ast., Asthma; AR. Allergic rhinitis; AC., Allergic conjunctivitis; CA., Contact allergy; OAS, Oral allergy syndrome; TS, Tape Strip; PMNs, Polymorphonuclear Cells; PBMCs, Peripheral Blood Mononuclear Cells; yo, years old; mo, months old; NA, missing data.

**Table S4.**

| **Target** | **Primer** | **Sequence (5'-3')** |
| --- | --- | --- |
| Mouse β-Actin  (NCBI gene ID: 11461) | Forward | CTCTTCCAGCCTTCCTTCCT |
|  | Reverse | AGCACTGTGTTGGCGTACAG |
| Mouse GAPDH  (NCBI gene ID: 14433) | Forward | CATCACTGCCACCCAGAAGACTG |
|  | Reverse | ATGCCAGTGAGCTTCCCGTTCAG |
| Mouse RPL13A  (NCBI gene ID: 22121) | Forward | CTGCTCTCAAGGTTGTTCGGCT |
|  | Reverse | CCTTCCGTTTCTCCTCCAGAGT |
| Mouse TSLP  (NCBI gene ID: 53603) | Forward | GCAAATCGAGGACTGTGAGAGC |
|  | Reverse | TGAGGGCTTCTCTTGTTCTCCG |
| Mouse IL-31RA  (NCBI gene ID: 218624) | Forward | ACACCGAGTTGGAGAGCCGTAT |
|  | Reverse | CTGTCCTCAGACCGATGTTCTC |
| Mouse IL-13  (NCBI gene ID:16163) | Forward | GCCAGCCCACAGTTCTAC |
|  | Reverse | AGACCACCAAGGCAAGC |
| Mouse IL-4  (NCBI gene ID: 16189) | Forward | CGAAGAACACCACAGAGAGTGAGCT |
|  | Reverse | GACTCATTCATGGTGCAGCTTATCG |
| Mouse IL-6  (NCBI gene ID: 16193) | Forward | TGGAGTCACAGAAGGAGTGGCTAAG |
|  | Reverse | TCTGACCACAGTGAGGAATGTCCAC |
| Mouse IL-10  (NCBI gene ID: 16153) | Forward | AGAAGCATGGCCCTGAAATCAAGG |
|  | Reverse | CTTGTAGACACCTTGGTCTTGGAG |
| Mouse IL-1β  (NCBI gene ID: 16176) | Forward | CAACCAACAAGTGATATTCTCCATG |
|  | Reverse | GATCCACACTCTCCAGCTGCA |
| Mouse IFNγ  (NCBI gene ID: 15978) | Forward | GGCCATCAGCAACAACATAAGCGT |
|  | Reverse | TGGGTTGTTGACCTCAAACTTGGC |
| Mouse IL-33  (NCBI gene ID: 77125) | Forward | CTACTGCATGAGACTCCGTTCTG |
|  | Reverse | AGAATCCCGTGGATAGGCAGAG |
| Mouse TNFα  (NCBI gene ID: 21926) | Forward | CTGTAGCCCACGTCGTAGC |
|  | Reverse | TTGAGATCCATGCCGTTG |
| Human β-Actin  (NCBI gene ID: 60) | Forward | CACCATTGGCAATGAGCGGTTC |
|  | Reverse | AGGTCTTTGCGGATGTCCACGT |
| Human GAPDH  (NCBI gene ID: 2597) | Forward | GTCTCCTCTGACTTCAACAGCG |
|  | Reverse | ACCACCCTGTTGCTGTAGCCAA |
| Human TRAP1  (NCBI gene ID: 10131) | Forward | CTCTGTGGAGACGGACATAGTC |
|  | Reverse | CTCCTTCTCTGATAGGCACTCG |
| Human HSP90AB1  (NCBI gene ID: 3326) | Forward | CTCTGTCAGAGTATGTTTCTCGC |
|  | Reverse | GTTTCCGCACTCGCTCCACAAA |
| Human HSP90B1  (NCBI gene ID: 7184) | Forward | GGAGAGTCGTGAAGCAGTTGAG |
|  | Reverse | CCACCAAAGCACACGGAGATTC |
| Human Hsp90AA1  (NCBI gene ID: 3320) | Forward | TCTGCCTCTGGTGATGAGATGG |
|  | Reverse | CGTTCCACAAAGGCTGAGTTAGC |
| Human TSLP  (NCBI gene ID: 85480) | Forward | TATCTGGTGCCCAGGCTATTCG |
|  | Reverse | TGAAGCGACGCCACAATCCTTG |
| Human IL-33  (NCBI gene ID: 90865) | Forward | GCCTGTCAACAGCAGTCTACTG |
|  | Reverse | TGTGCTTAGAGAAGCAAGATACTC |
| Human FLG  (NCBI gene ID: 2312) | Forward | GCTGAAGGAACTTCTGGAAAAGG |
|  | Reverse | GTTGTGGTCTATATCCAAGTGATC |

NCBI, National Center for Biotechnology Technology.
