# Supplementary material for: Topical application of the HSP90 inhibitor 17-AAG reduces skin inflammation and partially restores microbial balance: implications for atopic dermatitis therapy
Source: Sci Rep. 2025 Jul 1;15:21245. doi: 10.1038/s41598-025-05307-3 (PMC12216750; doi:10.1038/s41598-025-05307-3)
Supplement: Supplementary file 2 — Supplementary Material 2 [file 41598_2025_5307_MOESM2_ESM.pptx]

## Slide 1
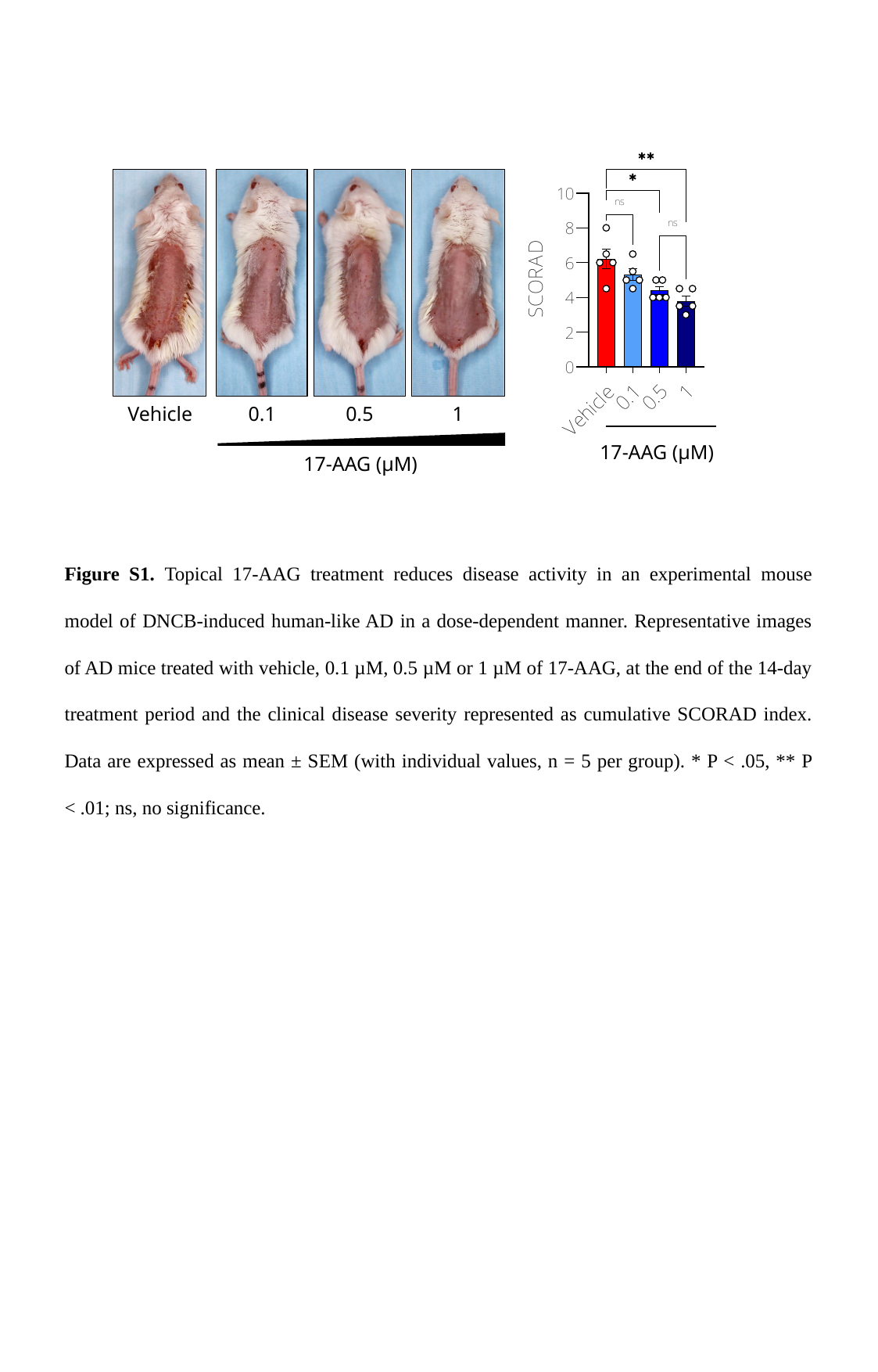

0.1
0.5
1
Vehicle
17-AAG (µM)
17-AAG (µM)
Figure S1. Topical 17-AAG treatment reduces disease activity in an experimental mouse model of DNCB-induced human-like AD in a dose-dependent manner. Representative images of AD mice treated with vehicle, 0.1 µM, 0.5 µM or 1 µM of 17-AAG, at the end of the 14-day treatment period and the clinical disease severity represented as cumulative SCORAD index. Data are expressed as mean ± SEM (with individual values, n = 5 per group). * P < .05, ** P < .01; ns, no significance.

## Slide 2
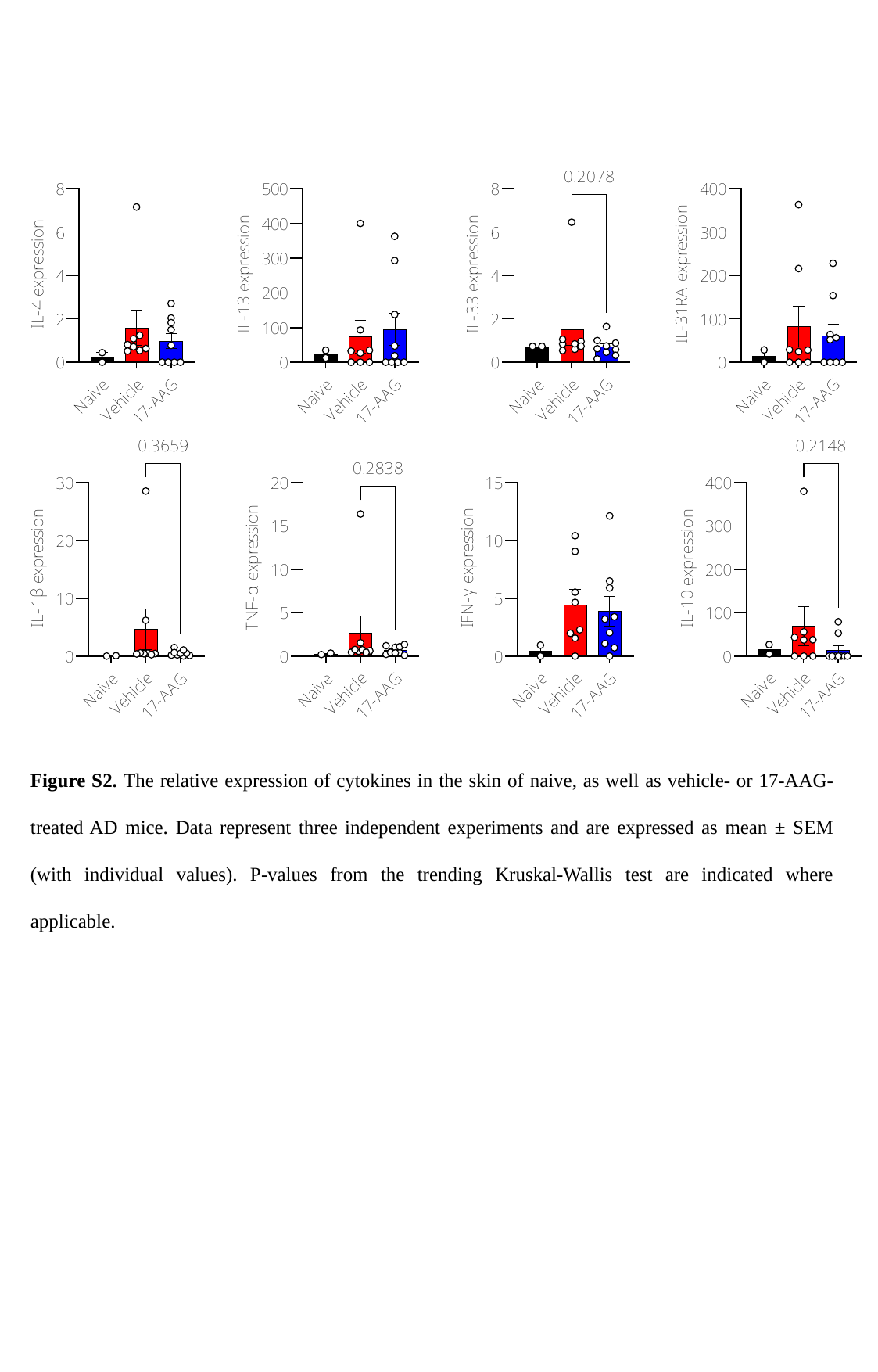

Figure S2. The relative expression of cytokines in the skin of naive, as well as vehicle- or 17-AAG-treated AD mice. Data represent three independent experiments and are expressed as mean ± SEM (with individual values). P-values from the trending Kruskal-Wallis test are indicated where applicable.

## Slide 3
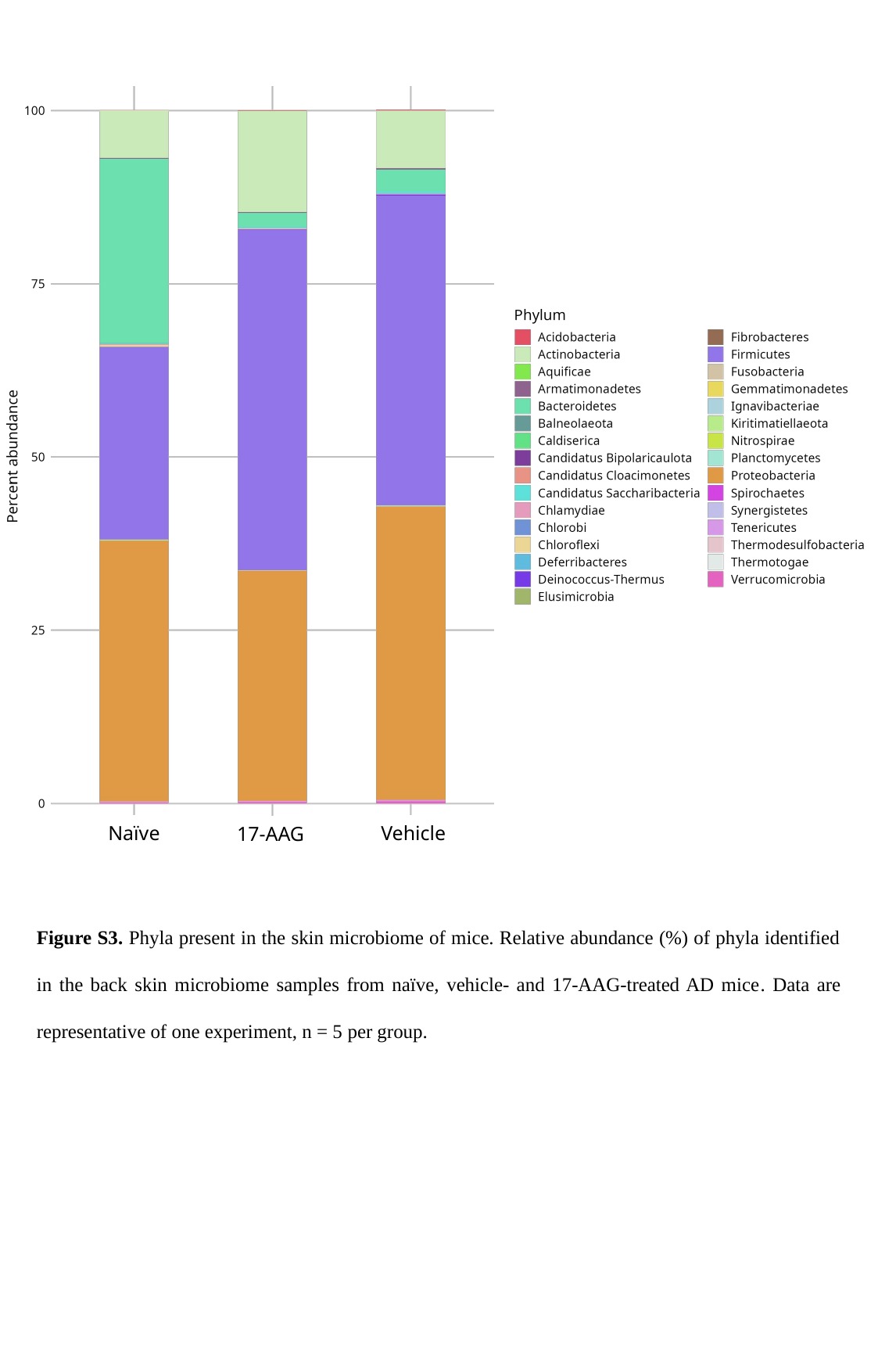

Naïve
Vehicle
17-AAG
Figure S3. Phyla present in the skin microbiome of mice. Relative abundance (%) of phyla identified in the back skin microbiome samples from naïve, vehicle- and 17-AAG-treated AD mice. Data are representative of one experiment, n = 5 per group.

## Slide 4
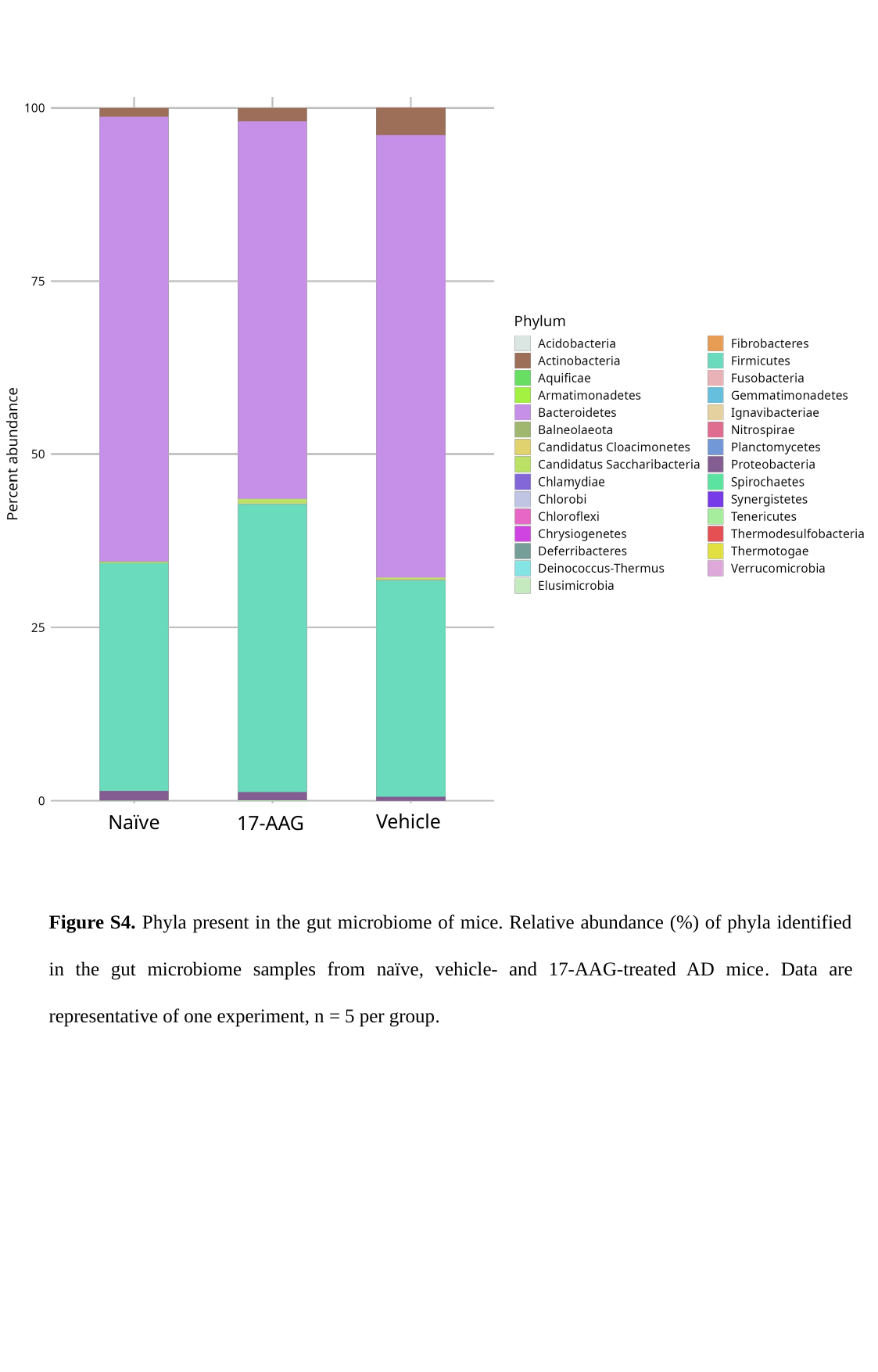

Vehicle
Naive
17-AAG
Naïve
17-AAG
Figure S4. Phyla present in the gut microbiome of mice. Relative abundance (%) of phyla identified in the gut microbiome samples from naïve, vehicle- and 17-AAG-treated AD mice. Data are representative of one experiment, n = 5 per group.

## Slide 5
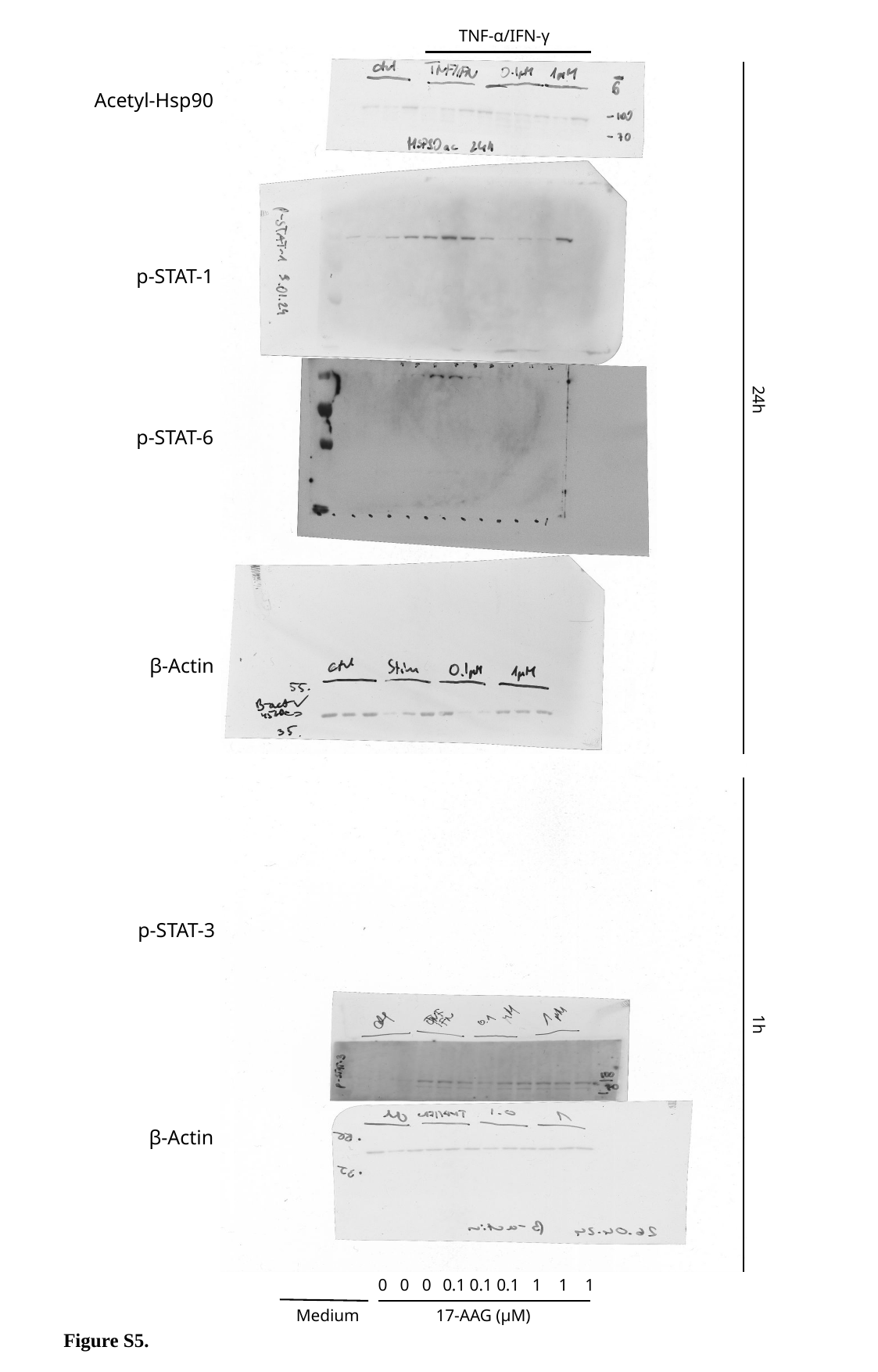

TNF-α/IFN-γ
Acetyl-Hsp90
p-STAT-1
24h
p-STAT-6
β-Actin
1h
p-STAT-3
β-Actin
0 0 0
0.1 0.1 0.1
1 1 1
17-AAG (µM)
Medium
Figure S5.
